# A semi-parametric Bayesian model for unsupervised differential co-expression analysis

**DOI:** 10.1186/1471-2105-11-234

**Published:** 2010-05-07

**Authors:** Johannes M Freudenberg, Siva Sivaganesan, Michael Wagner, Mario Medvedovic

**Affiliations:** 1 Laboratory for Statistical Genomics and Systems Biology, Department of Environmental Health, University of Cincinnati College of Medicine, 3223 Eden Ave. ML 56, Cincinnati OH 45267-0056, USA; 2 Mathematical Sciences Department, University of Cincinnati, Cincinnati, OH 45221, USA; 3 Division of Biomedical Informatics, Cincinnati Children's Hospital Medical Center, Cincinnati, Ohio 45229, USA

## Abstract

**Background:**

Differential co-expression analysis is an emerging strategy for characterizing disease related dysregulation of gene expression regulatory networks. Given pre-defined sets of biological samples, such analysis aims at identifying genes that are co-expressed in one, but not in the other set of samples.

**Results:**

We developed a novel probabilistic framework for jointly uncovering contexts (i.e. groups of samples) with specific co-expression patterns, and groups of genes with different co-expression patterns across such contexts. In contrast to current clustering and bi-clustering procedures, the implicit similarity measure in this model used for grouping biological samples is based on the clustering structure of genes within each sample and not on traditional measures of gene expression level similarities. Within this framework, biological samples with widely discordant expression patterns can be placed in the same context as long as the co-clustering structure of genes is concordant within these samples. To the best of our knowledge, this is the first method to date for unsupervised differential co-expression analysis in this generality. When applied to the problem of identifying molecular subtypes of breast cancer, our method identified reproducible patterns of differential co-expression across several independent expression datasets. Sample groupings induced by these patterns were highly informative of the disease outcome. Expression patterns of differentially co-expressed genes provided new insights into the complex nature of the ER*α *regulatory network.

**Conclusions:**

We demonstrated that the use of the co-clustering structure as the similarity measure in the unsupervised analysis of sample gene expression profiles provides valuable information about expression regulatory networks.

## Background

Examination of genome-wide patterns of gene expression levels is frequently used to characterize differences and similarities between biological samples at molecular level, and to elucidate underlying biological pathways and molecular networks. The analysis of gene expression profiles commonly focuses on either *differential *expression or *co*-expression [1]. In the former case, the goal is to identify genes whose expression level varies between two or more sample types or conditions. In contrast, co-expression (cluster) analysis is used to group together genes with similar expression patterns across different samples, and to group samples with similar global expression profiles.

Methods for co-expression analysis of gene expression data have been extensively researched, and numerous clustering algorithms have been developed and tested in this setting [2,3]. The clustering of both genes and samples using the same expression data is commonly termed as two-way clustering [4]. On the other hand, an entire class of unsupervised machine learning procedures has been developed for identifying bi-clusters (subsets of genes similarly expressed in a subset of samples) in gene expression data [3,5-8], and gene expression modules, which in addition to bi-clustering structure also incorporate information about gene expression regulation [9-12].

More recently, *differential co-*expression [13-15] has been used to characterize dysregulation of gene expression regulatory networks in prostate cancer [16], leukemia [17,18], or muscle growth [19]. In such analyses, genes which are co-expressed within one biological context (e.g. normal prostate tissue samples) but not within another context (e.g. prostate tumor samples) are said to be differentially co-expressed. These studies demonstrated that some of the known disease related-genes, which could not be identified by differential expression analysis, were actually differentially co-expressed [16,17,19]. A particularly straightforward example of such an analysis comes from gene expression comparisons of developing muscle tissue in a bovine animal model (Wagyu cattle) and the double-muscled model (Piedmontese cattle) expressing a version of the myostatin (MSTN) transcription factor known to carry the causal mutation for the observed phenotype. Since the expression of the myostatin gene itself is not affected by the mutation, differential expression analysis fails to identify it as being functionally relevant. However, this gene is implicated through a differential co-expression analysis since the functional version (in Wagyu model) is co-expressed with its regulatory targets such as MYL2 while the non-functional version (in Piedmontese cattle) is not [19]. Differential co-expression analysis methods to date require the definition of biological contexts within which the co-expression is to be compared.

Here we present a novel probabilistic approach for uncovering contexts (i.e. groups of samples) with specific co-expression patterns and sets of genes that are differentially co-expressed between such contexts. Our probabilistic differential co-expression infinite mixture (DCIM) model implicitly defines a new similarity measure for biological samples based on the similarity of the gene co-expression structure within each sample. Two samples are deemed similar according to this measure if the same groups of genes are co-clustered in both samples regardless of the overall similarity of the gene expression patterns in the classic sense, such as those implied by high correlation and small Euclidean distance. This makes our procedure fundamentally different from currently used clustering and bi-clustering methods. To the best of our knowledge, this is the first time that patterns of co-expression derived from gene expression data, and not gene expression levels themselves are being used to cluster biological samples, and the first framework for unsupervised analyses of differential co-expression, where co-expression is defined in such general terms.

Our DCIM model is based on Bayesian semi-parametric Dirichlet process mixtures [20], also referred to as the infinite mixture model [21]. This methodology has been applied in clustering gene expression data [22,23] and has been shown to effectively circumvent the difficult issue of specifying or estimating the "correct" number of clusters [23-25]. The context specificity of the gene co-expression patterns is defined as in the context-specific infinite mixture (CSIM) model [26]. We have previously shown that *a-priori *knowledge of partitions of samples into contexts with differential gene co-expression patterns can be exploited to improve the functional coherence of resulting gene clusterings [26] and transcriptional modules [27]. Here we expand this model to *de-novo *partitioning of samples into contexts of differential co-expression. To facilitate the *de-novo *search for contexts, we impose additional Dirichlet process-like priors on the membership of samples in different contexts. The use of infinite mixtures allows us to avoid specifying the number of global and local gene expression clusters as well as the number of contexts. Co-expression relationship and co-memberships in the same context are estimated by integrating over all possible values of these key parameters.

In the case of breast cancer, studies of genome-wide patterns of gene expression levels have lead to the discovery of distinct molecular subtypes differing in clinical, histological, and molecular characteristics, as well as treatment response and disease outcome [28-31]. They point to a diverse etiology of the disease with distinct molecular signatures involving numerous biological processes. Some of these findings are currently used in clinical trials aiming to improve patient prognosis and treatment [32]. Using the new methodology, we revisit the problem of identifying molecular subtypes of breast cancer. We find that the patient groupings induced by the differential co-expression strongly predict disease outcome. Differentially co-expressed genes as well as the patterns of differential co-expression are highly reproducible across independent expression datasets. The differential co-expression 'signal' identified in our analysis is complementary to other predictive parameters such as estrogen receptor (ER) status, lymph node (LN) status, and AURKA expression as well as the 'signals' contained in the clusters of samples created using traditional similarity measures.

## Results

### Context-specific infinite mixture model

**Figure 1:**
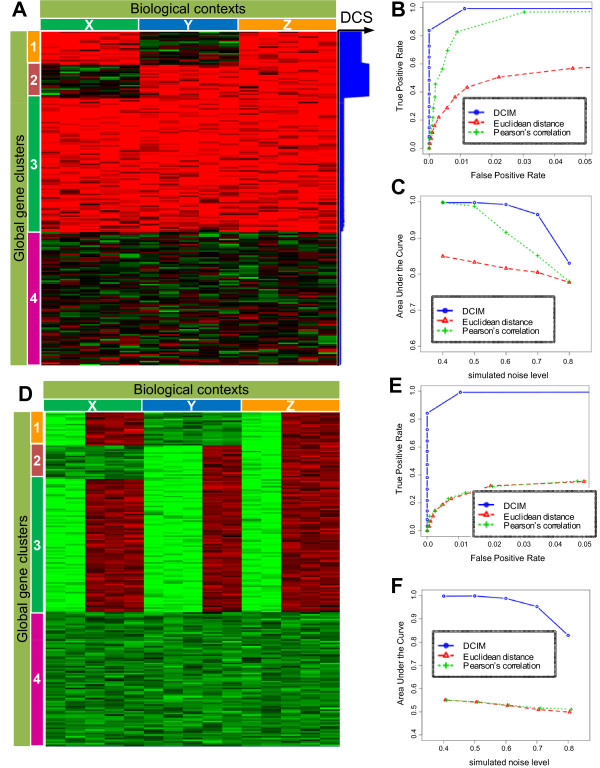
**Simulation study results**. **A) **Genes grouped into global gene clusters, marked 1-4, are further grouped locally within respective biological contexts, marked X, Y, and Z. Conversely, biological samples are in the same biological context if they have the same groupings of co-clustered genes. Differential co-expression score (DCS) is displayed in the right hand side-panel. **B) **Average ROC curves were obtained for repeatedly simulated data with noise levels ranging from *σ *= 0.4 0.8, with *σ *= 0.5 displayed here, by averaging the FPRs (incorrectly co-clustered pairs of samples) and TPRs (correctly co-clustered pairs) for each distinct tree cut level. **C) **To summarize ROC curves over all simulations at a given noise level *σ*, we compute the area under the curve (AUC) for each simulation and plot the average AUC against *σ*. **D) **The context structure is same as in **A**, but expression patterns within each context are modified. **E) **and **F) **same as **B **and **C **but for the clustering structure in **D**. Groupings based on traditional similarity measures no longer corresponded to the underlying context structure. DCIM algorithm continues to correctly identify the underlying contexts.

The DCIM model is based on the assumption that global gene clusters, consisting of genes with similar expression patterns across all samples, are grouped further into local clusters within each context consisting of samples with identical co-expression structure. In Figure 1A samples (i.e. columns) are organized into three contexts, and genes (i.e. rows) form four global clusters. Within context X, global clusters 1 and 3 are further grouped into a single local cluster and global clusters 2 and 4 are grouped into another local cluster. Consequently, within context X all gene expression profiles form only two local clusters. Similarly, within context Y, gene clusters 1 and 4 form a local cluster and gene clusters 2 and 3 form a local cluster. Since the local clustering of genes is different between groups of samples X and Y, these two groups form two different contexts. As a result, each context is characterized by a unique co-clustering structure of genes.

**Figure 2:**
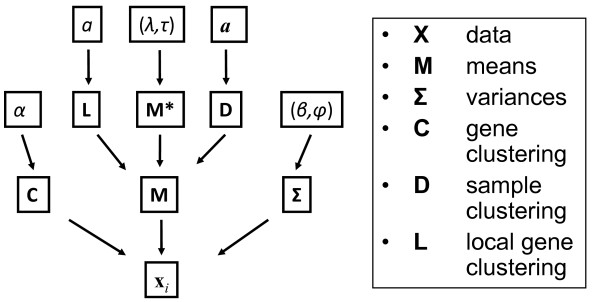
**Directed acyclic graph describing the DCIM computational model**. Nodes represent random variables and edges indicate dependencies between nodes such that each random variable is conditionally independent of its non-descendents given its parent nodes (local Markov property).

The DCIM model is specified in terms of a Bayesian Network [33]. A directed acyclic graph (DAG) specifying conditional dependences in terms of the directed Markov property is shown in Figure 2. The local probability distributions for the key variables specifying the allocation of genes into global clusters (*C*), the allocation of global clusters into local clusters within each context (**L**) and the allocation of samples into different contexts (*D*) are given in terms of the priors derived from the respective Dirichlet processes. The joint posterior distribution of all parameters specified by the Bayesian Network is estimated using a Gibbs sampler. Marginal posterior distributions of the three key allocation variables (*C*, *D*, **L**) are summarized in terms of the posterior pair-wise probabilities (PPPs) of global and local co-expression for each pair of genes and the PPPs of belonging to the same context for each pair of samples.

Using the local PPPs of co-expression derived from the model, we apply a heuristic algorithm to search for differences between the local gene clusterings and identify genes that are differentially co-expressed between two contexts. The higher the resulting differential co-expression score (DCS) is for a gene, the higher the likelihood that this gene's co-clustering partners are different between the two contexts. In Figure 1A, genes with high DCS between contexts X and Y+Z contexts are indicated by the thick blue bar on the right-hand side of the heatmap. Genes in cluster 1 distinguish context Y from contexts X and Z, genes in cluster 2 distinguish context X from contexts Y and Z. Taken together, they define all three contexts.

Technical details are provided in Methods and Additional file 1; Supplemental Methods (support website http://eh3.uc.edu/gimm/dcim). Computational procedures for fitting the model are implemented in the R package *gimmR *which can be downloaded freely from http://ClusterAnalysis.org. Using our DCIM algorithm we also performed a large scale cluster analysis and functional annotations of the results for virtually all human, mouse and rat GeoDataSets [34]. Results of these analysis can be accessed through Genomics Portals http://GenomicsPortals.org[35].

### Recovery of simulated contexts

We first evaluate our method using a series of simulated datasets at different noise levels with the data structure shown in Figure 1A. Receiver Operating Characteristics (ROC) curves summarizing the true and false positives rates of co-clustered pairs of samples for each clustering imply favorable performance of the DCIM algorithm in comparison to traditional hierarchical clustering methods (Figure 1B). The average area under the ROC curve (AUC) is consistently higher for our DCIM algorithm when compared to traditional clustering algorithms (Figure 1C), indicating a higher level of precision in reconstructing sample grouping across the whole range of noise levels.

To further accentuate the conceptual difference between the sample groupings based on our context-building algorithm and traditional similarity measures, we modified the simulation procedure (Figure 1D) leaving the co-expression structure unchanged but modifying the relative expression levels. For example, all "samples" in the first context still have identical co-expression structure, but the mean expression profile of the first two "samples" is different from the mean expression profile of the last three "samples". As expected, groupings based on traditional similarity measures no longer correspond to the underlying context structure. In contrast, the DCIM algorithm continues to correctly identify the underlying contexts (Figures 1E and 1F). These results indicate that, in general, DCIM can be expected to produce groupings of biological samples that will be different from the groupings constructed using the traditional similarity measures.

This clear difference between DCIM and other clustering methods was also evident when we re-analyzed the e bovine animal model data [19] comparing wild type cross (Wagyu × Hereford) and double muscle cross (Piedmontese × Hereford) at 10 developmental time points (Additional file 1; Figure S1.A). Here, much like genes in clusters 3 and 4 in Figure 1, one transcription factor (myostatin) has similar expression levels across all samples. However, its transcriptional targets such as MYL2 are differentially co-expressed at certain developmental stages due to the mutated myostatin in Piedmontese cattle (like clusters 1 and 2 Figure 1). The resulting two top level sample contexts split into pre-natal and post-natal time points. In contrast, simple hierarchical clustering methods (Euclidean distance, Pearson correlation) consistently grouped the same time points (e.g. Piedmontese and Wagyu cattle at 280 days) as pairs of most similar samples, but no obvious separation of time points (Additional file 1; Figure S1.B-D). The comparison of top DCS genes identified in our unsupervised analysis for Piedmontese vs. Wagyu cattle and the 85 DE genes identified in the original paper [19] showed statistically highly significant overlap(Fisher *p*-values 1.6 × 10^-12 ^and 6.7 × 10^-20 ^for the top 85 and top 200 DCS genes). All but one DE gene (CYP4B1) had above median DCS. We then repeated the functional analysis reported by Hudson *et al*. [19] and found similar significantly enriched categories related to muscle structural components. Both findings indicate that the differentially co-expressed genes indeed are likely to be transcriptional targets of myostatin.

Similarly to traditional clustering procedures, traditional bi-clustering procedures applied to data with a co-clustering structure as in Figure 1D should also fail to reconstruct underlying contexts. For example, samples with different mean expression profiles in context Z in Figure 1D have low pair-wise correlation and relatively high Euclidean distance. Consequently, they should not be grouped together to form bi-clusters. Since bi-clustering procedures are not designed to cluster all samples, we cannot construct equivalent ROC curves. Instead, we attempted to make this point by performing bi-clustering analysis of two "easy" (low-noise) examples from our simulation study. Results are shown in Additional file 1; Figure S2. The performance of the two-way hierarchical procedures (Euclidean distance, Pearson's correlation and DCIM) on these two examples was as expected (Additional file 1; Figure S2.A and C). Also as expected, all five bi-clustering methods tested as implemented in the Biclustering Analysis Toolbox v2.2 [36] produced groupings of samples that did not correspond to context structure for the scenario in Figure 1D (Additional file 1; Figure S2.D). While the behavior of bi-clustering procedures generally was peculiar for even the simple clustering structure, only one of the methods (BiMax) produced reasonably shaped bi-clusters (after adjusting the discretization parameter to match the simulated clustering structure; Additional file 1; Figure S2.B). However, the point of the comparisons shown here is not to claim that DCIM is "better" than traditional clustering and bi-clustering procedures, but to show that it produces sample groupings based on an implicit similarity measure which by design is "different" from traditional similarity measures.

### Identifying molecular subtypes in breast cancer gene expression data

**Figure 3:**
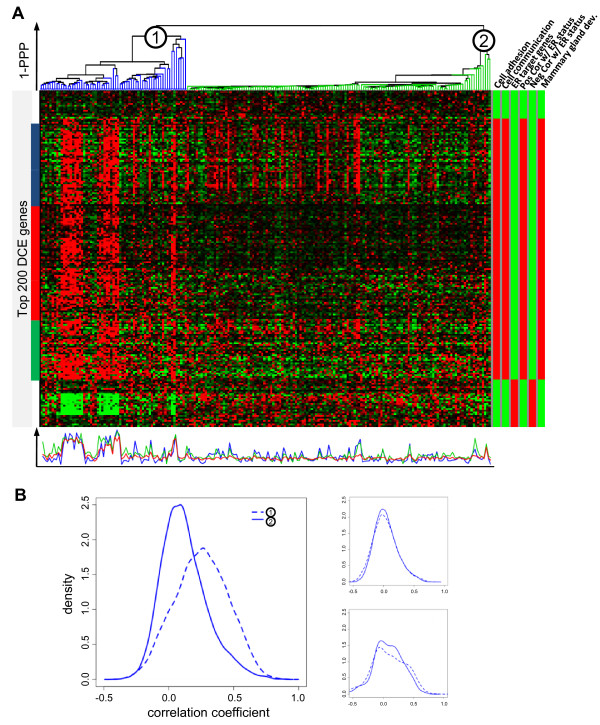
**DCIM derived contexts and related differentially co-expressed genes**. DCIM was used to identify contexts and differentially co-expressed genes in a breast cancer dataset [37]**A) **Hierarchical clusterings of patients based on differential co-expression PPPs and the heatmap of 200 most differentially co-expressed genes between two contexts marked (1) and (2). The bottom panel shows the average expression profile for the three global gene clusters marked in the heatmap with corresponding color sidebars. The right-hand panel shows significantly enriched functional categories for these genes as determined by CLEAN [55] where red indicates the corresponding cluster is significantly enriched by the category and green indicates no significant cluster enrichment. Complete CLEAN results for all possible gene clusters can be interactively browsed using the FTreeView software at the support website http://eh3.uc.edu/gimm/dcim. **B) **Empirical distribution of all pairwise gene-gene correlation coefficients (Pearson correlation) for the 154 genes marked by the left sidebar in **A**. The top right plot shows correlations for 154 randomly selected genes in the same two contexts while the bottom right plot shows correlations for the same genes but with randomly reassigned context labels.

**Figure 4:**
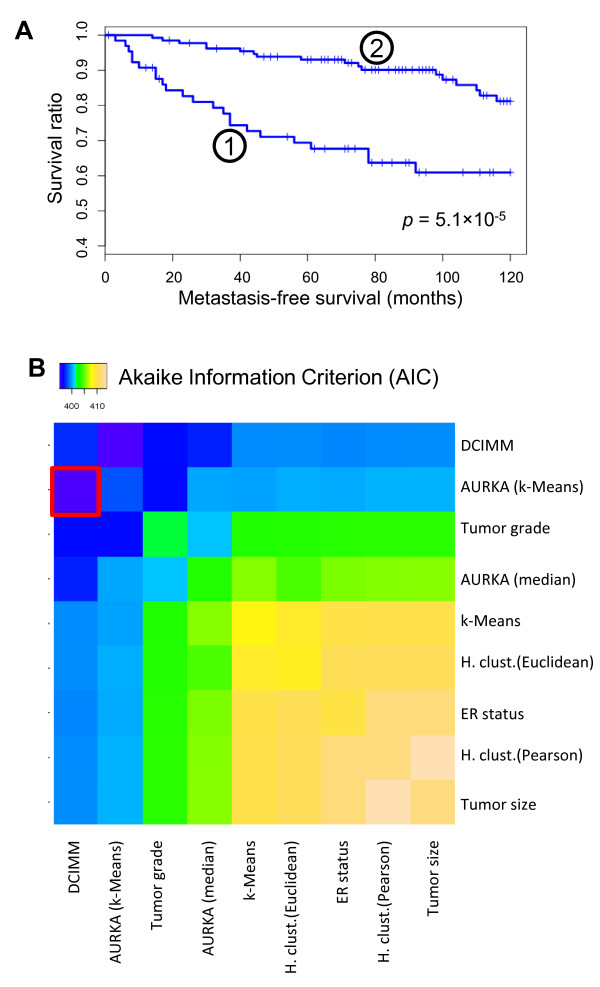
**DCIM derived contexts in breast cancer data are predictive of patient survival**. DCIM was used to identify the top two contexts marked (1) and (2) in Figure 3 and survival analysis was performed. **A) **Kaplan-Meier curves for the two contexts. **B) **Clinical, molecular, and computational parameters and their pairwise combinations were used to fit one-parameter and two-parameter Cox regression and the model fit was assessed using the Akaike Information Criterion (AIC). The model combining DCIM contexts and AURKA expression defined patient groups best predicts patient survival (red box) http://eh3.uc.edu/gimm/dcim.

We now examine the biological importance of uncovering differential co-expression structure by performing alternative molecular sub-typing of breast cancer samples in a recent breast cancer gene expression dataset [37]. Figure 3A shows the resulting hierarchical clustering of patient samples based on PPPs and the expression patterns of the 200 most differentially co-expressed genes. Two distinct sample groups or contexts are noticeable. A closer examination of the samples in two dominant contexts revealed a high correlation with key clinical parameters such as estrogen receptor (ER) status, tumor grade, and tumor size (Additional file 1; Table S1). The membership in two contexts was also highly predictive of the disease outcome as indicated by Kaplan-Meier survival curves (Figure 4A) (logrank *p*-value = 5.1 × 10^-5^) and statistically significant differences in 10 year survival rates (60.9% vs. 81.2%, *p*-value = 3.4 × 10^-3^). Traditional similarity/distance measures induced considerably different sample groupings (Additional file 1; Table S2) which had little or no correlation with the disease outcome (Table 1).

**Table 1:** Associations with the survival outcome using different clinical, molecular, and computational methods (Schmidt *et al*. dataset [37]).

Parameter			Size of patient groups		logrank *p*-value
			
			poor survival	favorable survival	
Clinical	Tumor size (≤ 2 cm, >2 cm)		88	112	0.17
	
	Tumor grade (G1, G2/G3)		165	35	3.7 × 10^-3^

Molecular	ER status		38	162	0.12
	
	AURKA expression	<, > median	100	100	4.1 × 10^-3^
		
		*k*-Means (*k *= 2)	62	138	8.7 × 10^-5^

Computational	Hierarchical clustering	Pearson correlation	71	129	0.16
		
		Euclidean distance	18	182	0.037
	
	*k*-Means clustering		64	136	0.043
	
	*DCIM*		*65*	*135*	*5.1 × 10*^-5^

### Differentially co-expressed genes

The functional analysis of the 200 genes most differentially co-expressed between the two major contexts revealed enrichment for genes both positively and negatively associated with ER status (Figure 3A). Genes negatively associated with ER status were tightly co-regulated within the "poor-prognosis" samples in context 1, but showed no co-expression within context 2. This cluster was also enriched for ER*α *regulatory targets as established in recent ChIP-chip experiments [38]. Genes positively associated with ER status are also tightly co-regulated, forming a large cluster (clusters marked by blue, red and green bars combined) within context 1. These same genes are generally less co-regulated (red cluster) or not at all co-regulated (blue and green clusters) within context 2. This combined cluster was also enriched for *Cell Adhesion, Cell Communication, and Mammary Gland Development *genes (Figure 3A). These differential co-expression patterns are reflected in the distribution of pairwise correlations shown in Figure 3B. Within context 1, the Pearson correlation coefficient between gene pairs is significantly higher than within context 2. Complete results of functional analysis for the 200 most differentially co-expressed genes are provided in Additional file 2; Table S9.

As in the second scenario of our simulation study (Figure 1D), sample groupings based on differential co-expression of these 200 genes with the highest DCS were considerably different than sample groupings generated by traditional similarity measures on these same genes. Furthermore, the differences in disease outcomes were much smaller for the sample groupings generated by the traditional hierarchical clustering methods and *k*-means algorithm (Additional file 1; Table S3). This indicates that our method not only identifies functionally important genes, but the implicit similarity measure based on the differential co-expression is necessary to optimally utilize expression patterns of these genes in predicting the disease outcome.

### Comparison to other outcome predictors

We compared the strength of association between disease outcome and the patient groupings induced by the DCIM algorithm to several alternative groupings based on important clinical and molecular parameters, as well as unsupervised clustering of patient samples based on the traditional similarity measures (Table 1). Among the parameters with statistically significant correlation with disease outcome were tumor grade and aurora kinase A (AURKA) gene expression, a proliferation associated gene shown to be a powerful predictor of survival in breast cancer [39]. Tumor size and ER status did not yield patient groups significantly different with respect to the disease outcome for this dataset. Given the high level of enrichment of ER status related genes among differentially co-expressed genes, it is particularly interesting that in this dataset ER status on its own was not strongly associated with the disease outcome. This indicates that the expression patterns of genes identified based on the **differential expression **between ER+ and ER- samples can be more predictive of the disease outcome in the context of **differential co-expression **analysis than ER status is on its own. Among the unsupervised computational methods we compared, the *k*-Means algorithm and Euclidean distance based hierarchical clustering resulted in patient groups with marginally statistically significant differences in disease outcome. The unsupervised analysis based on our differential co-expression measure yields the highest statistical significance for differences in survival between sample groupings.

To assess the independent contribution of the differential co-expression signature to the predictive models based on other variables, we systematically evaluated the benefit of combining two classification methods using Cox regression. We found that the model significantly improved when including DCIM based classification as compared to using any other variable alone. In particular, the model combining DCIM and AURKA expression had the lowest overall Akaike Information Criterion (AIC) (395.3) indicating the best model fit (Figure 4B).

### Reproducibility of differential co-expression signature across independent datasets

**Figure 5:**
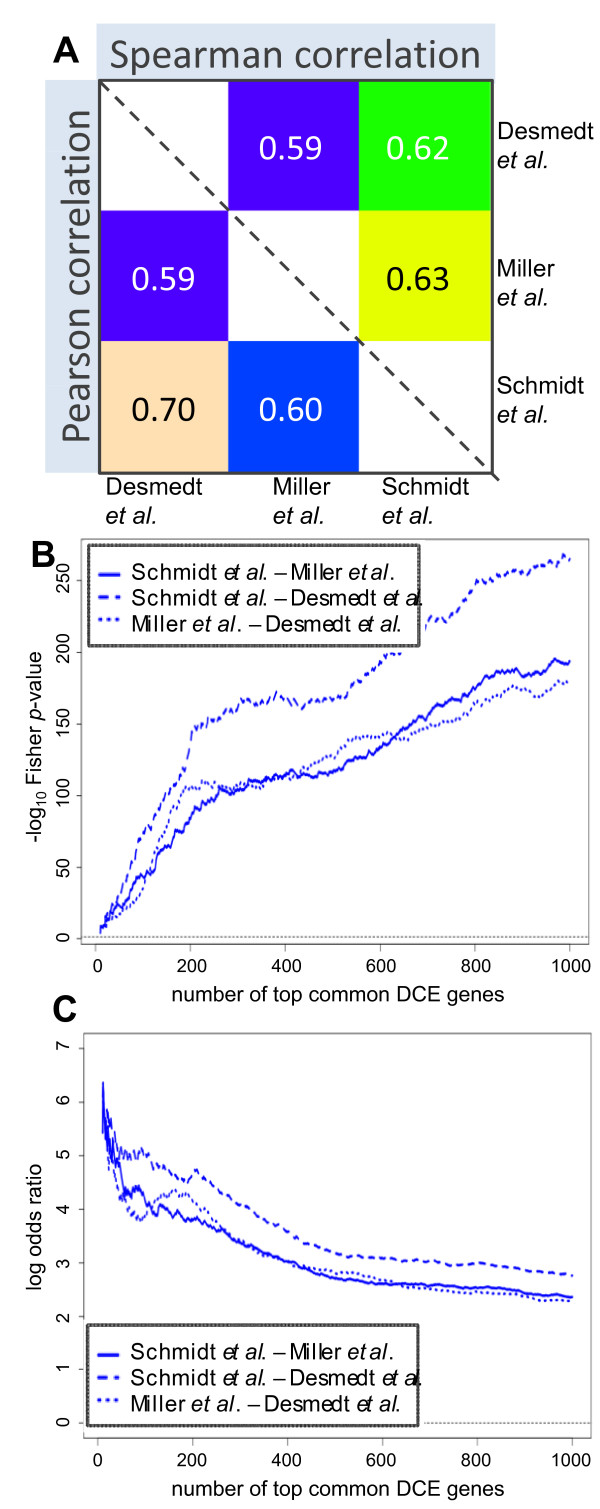
**Reproducibility of differential co-expression scores (DCS) in independent datasets**. **A) **Pair-wise correlations between DCS for all genes shows high correlation for both Pearson's and Spearman's correlation coefficients **B) **Statistical significance of pair-wise overlaps between lists of top 1-1000 most differentially co-expressed genes shows high level of concordance. All three curves are peaking above Fisher's Exact Test p-value of 10^-150 ^**C) **Relative pair-wise overlaps in terms of log-odds ratios show non-trivial level of overlaps (log-odds ratio > 2) throughout the range (1 to 1000 most differentially co-expressed genes).

**Figure 6:**
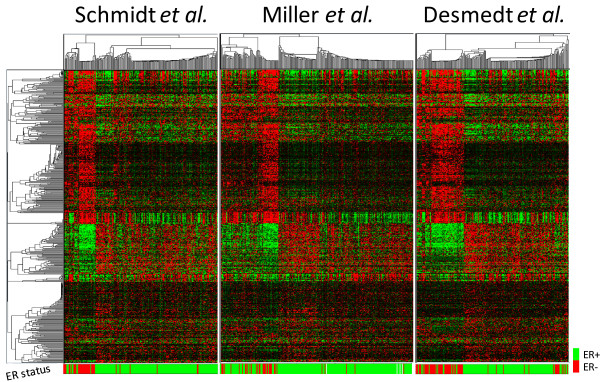
**Heatmaps of top 500 DCS gene signature in three breast cancer studies**. Expression patterns are remarkably consistent across different datasets. DCIM sample clusterings are highly correlated with ER status. Gene expression profiles and functional annotations can be accessed and examined interactively at the support web-site http://eh3.uc.edu/gimm/dcim.

The reproducibility of results was assessed by repeating the analysis on two additional breast cancer datasets [40,41]. The high correlations between DCS measures (Figure 5A) and the highly significant overlaps between the lists of genes with highest DCS (Figure 5B and 5C) for different datasets indicate the reproducibility of differential gene co-expression. Using information from all three datasets, we constructed a "differential co-expression signature set" by selecting a list of the 500 common genes that had a top-ranking DC score in each of the three datasets. Using only these genes to re-analyze all three datasets the DCIM algorithm yielded remarkably consistent patterns of differential co-expression (Figure 6). Similar results were obtained when using the top 200 DCE genes shown in Figure 3A (Additional file 1; Figure S3). Despite the fact that the Miller *et al*. dataset [40] also contained samples from lymph node positive patients (Additional file 1; Table S4), the overall gene expression patterns in the two contexts were concordant to expression patterns in the other two datasets. The lymph node status was in this case the strongest single predictor of the disease outcome, but the co-expression signal together with the lymph node status provided for the best model fit in explaining the disease outcome among all 2-predictor combination (Additional file 1; Figure S4).

### Meta-analysis based on the differential co-expression signature

**Figure 7:**
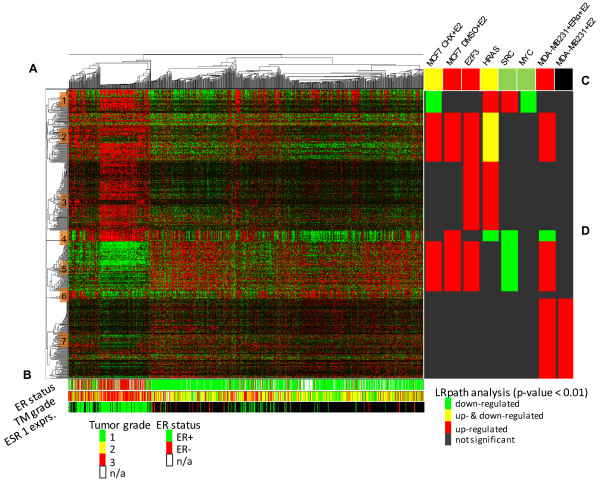
**DCIM analysis of the top 500 DCS signature in the combined dataset**. **A) **Expression patterns are consistent with the results for the individual datasets. **B) **ER status, tumor grade, and the expression levels of the ER*α *transcription factor. DCIM sample groupings are highly correlated with all three variables. **C) **LRpath analysis [56]of enrichment by the genes up- and down-regulated by E2 and oncogenic pathways genes for the whole DCE signature. **D) **Separate LRpath analysis of enrichment for seven distinct sub-clusters. Gene expression profiles and functional annotations can be accessed and examined interactively at the support website http://eh3.uc.edu/gimm/dcim.

The predictive potential of the differential co-expression signature was then tested in the analysis of a 'super'-set (989 samples) comprised of the three independent datasets described above and additional three studies [30,42,43]. Using the DCIM algorithm to cluster samples based on the 500 DC signature genes (Figure 7A), we again observe patient groupings with significantly different disease outcomes (logrank *p *= 3.8 × 10^-3^), highly significant correspondence to the groupings found when analyzing the data sets individually (Additional file 1; Table S5, odds ratio = 15.9, Fisher *p*-value = 1.6 × 10^-77^), and high correlation to ER status and tumor grade (Figure 7).

### Estrogen receptor alpha and oncogenic pathway analysis

Given the strong correlation of the contexts induced by our algorithm and the ER status of the samples, we examined the differentially co-expressed gene clusters in the context of ER*α *transcriptional targets. We distinguished four different sets of genes dysregulated by ER*α*: Primary and overall transcriptional effects elicited by stimulating the ER positive MCF-7 breast cancer cell line with estradiol [44], and effects elicited after stimulating the ER negative MDA-MB-231 breast cancer cell line with estradiol with and without re-expressing ER*α *[45]. We also examined correlations with the transcriptional targets of four oncogenes (E2F3, HRAS, SRC and MYC) elicited after transfecting cultured primary human mammary epithelial cells with adenovirus expressing one of the four oncogenes [46]. The overall DC signature gene set was enriched for genes up-regulated in E2 treated, cycloheximide-pretreated and not pre-treated MCF-7 cells (CHX+E2 and E2), E2 treated MDA-MB-231 after ER*α *re-expression, and after HRAS and E2F3 induction. It was also enriched for genes downregulated in CHX+E2 treated MCF-7 cells, and after HRAS, SRC and MYC induction (Figure 7C).

By splitting the signature into 7 clusters of co-expressed genes we further refined the correlation between different expression patterns within the DC signature and these six biological systems (Figure 7D). For example, both Clusters 2 and 5, despite their opposite expression patterns, were enriched by genes upregulated by estradiol treatment in the presence of functional ER*α*, while Cluster 3 was enriched by genes upregulated in two oncogenic pathways (E2F3 and RAS), but not by estradiol. Clusters 1 and 2, which showed similar expression pattern in the left context, but not in the right context, were enriched by primary estrogen targets were regulated in opposite direction by E2 treatment of MCF7 cell line.

## Discussion

We have developed an analytical procedure for unsupervised differential gene co-expression analysis. The DCIM algorithm produces two-way hierarchical clusterings of all genes and samples. The implicitly defined similarity measure between biological samples is based on the similarities in the clustering structure encoded in the DCIM model. As demonstrated in the simulation study and analysis of the bovine myostatin data, this similarity measure is fundamentally different from traditional measures of similarity between gene expression profiles used by clustering and bi-clustering algorithms to date.

Breast cancer sample groupings based on differential co-expression were more strongly correlated with the disease outcome than the sample groupings produced by traditional clustering techniques. Differentially co-expressed genes identified by our algorithm are functionally related to the etiology of breast cancer and are reproducible across independent breast cancer datasets. A large portion of these genes are directly regulated by the ER*α *transcription factor and reside at the intersection of various oncogenic pathways. Our analysis establishes the ER*α *status as the dominant factor defining contexts of differential co-expression in breast cancer samples.

The complex sets of transcriptional signatures recovered by our algorithm separating ER positive and ER negative breast cancer samples can be explained by the complex nature of ER regulation of its transcriptional targets. This regulation is highly context-specific and it is generally believed to be driven to a large extent by the complex interactions of ER with different co-factors [47]. The most striking difference in ER transcriptional regulation in two different biological contexts is demonstrated by the opposite effects its activation has in ER positive cell cancer lines such as MCF-7, where it stimulates proliferation and growth, and in ER negative cell cancer lines, where re-expressing ER*α *facilitates the anti-proliferative effects of estradiol [45]. In breast cancer samples with a functional ER*α *gene, sets of differentially co-expressed genes with distinct expression patterns are regulated through ER*α *interactions with different co-factors. In samples without the functional ER*α *gene, these genes are "less" regulated and their expression patterns are simplified into three dominant expression patterns indicated in Figure 7 by cluster numbers 1-4 ("up"), 5-6 ("down") and 7 ("unchanged").

Gene expression profiling of breast cancer samples has been used to derive numerous distinct, but often overlapping gene lists that are predictive of the disease outcome [32]. On the other hand, it has been shown that the general "proliferation" signature underlies predictive ability of many of such lists [48] and that gene expression profile of a single proliferation marker gene (AURKA) can serve as a surrogate for the predictive ability of such lists [39]. In our analysis, DCE-based classification of samples into different contexts was complementary to other clinical, pathological and molecular predictors including AURKA gene expression. We also found that our 500 gene DCE signature has a significant overlap with the experimentally derived list of "intrinsic genes" [31] (Additional file 1; Table S6). The "intrinsic genes" signature consisting of genes with high between-to-within-tumor ratio of expression variability, has served as a gold standard for molecularly classifying breast tumors [31,49,50], and has also been shown to contain predictive ability independent of the clinical parameters.

## Conclusions

The biological relevance of both sample groupings and differentially co-expressed genes identified in our analysis suggest that our DCIM framework can produce useful new insights into the gene expression regulatory networks.

## Methods

### Differential co-expression infinite mixture (DCIM) model

Suppose **X **is the *N *× *M *expression data matrix where *x*_*ij *_is the expression level of gene *i *in sample *j*. Accordingly, **x**_*i *_= (*x*_*i*1_, *x*_*i*2_, ..., *x*_*iM*_) is the *global expression profile *for gene *i *and **x**_*j *_^T ^= (*x*_1*j*_, *x*_2*j*_, ..., *x*_*Tj*_)^T ^is the expression signature for sample *j*.

*C *= (*c*_1_, *c*_2_, ..., *c*_*N*_)^T ^is the vector of gene allocation variables assigning genes to underlying expression profiles; *c*_*i *_= *q *means that expression profile ***x***_*i *_is generated by the underlying pattern *q *represented by the *M*-dimensional multivariate normal distribution *N*_*M*_(*μ*_*q*_, *Σ*_*q*_). Groups of genes generated by the same distribution form a *global gene cluster*.

Likewise, D = (*d*_1_, *d*_2_, ..., *d*_*M*_) is the *M*-dimensional vector of allocation variables assigning each sample to a context; *d*_*j *_= *r *means that expression signature **x**_*j *_^T ^belongs to context *r*. Global expression patterns which are indistinguishable within a context are further grouped into *local clusters*. The local gene clustering structure is represented by *the *matrix **L **where *l*_*qr *_= *t *means that, within context *r*, global cluster *q *is grouped into local cluster *t*.

The joint distribution of data and model parameters is specified by a Bayesian network. The Directed Acyclic Graph (DAG) in Figure 2 specifies conditional independencies in terms of the directed Markov property [33]. Given the DAG and conditional probability distributions of each node given its parents, the joint probability distribution is

where *M *= {*μ*, ..., *μ*_*Q*_} and Σ = {Σ_1_, ..., Σ_*Q*_} are the mean vectors and variance-covariance matrices defining the expression patterns **x**_*i*_. The prior probability distributions for the random variables defining the global gene clustering **C**, local gene clustering **L**, and sample to context assignment **D **are derived from the Dirichlet process priors and do not require specification of the number of groups [23,26]. The prior probability that a sample *j *will be placed in already existing context *r *is  while the prior probability of *j *being placed in a new context is  where *n*_-*j*, *r *_is the number of samples currently in context *r *without *j*.

### Fitting the model

Inference about gene clusters and sample contexts is based on the marginal posterior distribution of parameters **C**, **L**, and **D**. These distributions are derived from the joint posterior distribution of the model parameters given data *p*(*C*, *D*, *L*, *M*, M*, Σ, *α*, *β*, *ϕ*, *a*, a, *λ*, *τ*|*X*) which is estimated using a Gibbs sampler [51]. The Gibbs sampler iteratively draws values from the conditional posterior probability distributions for each random variable in the model given all other variables and the data. The resulting Markov Chain converges to the joint posterior distribution. In particular, the posterior conditional probability for placing sample *j *into existing context *r *is given by

Estimated posterior marginal distributions of **C**, **L**, and **D **are summarized by calculating posterior pair-wise probabilities of co-groupings as the proportion of Gibbs sampler cycles in which two genes or samples were grouped together. Hierarchical clusterings of genes and samples are created by using PPPs as the similarity measure and applying the average linkage agglomeration method. All prior and conditional posterior probability distributions that specify the model and facilitate the estimation of the posterior distribution of model parameters are provided in Additional file 1; Supplemental Methods.

### Differential co-expression score

Given two contexts, we consider a pair of genes differentially co-expressed (DCE), if they are co-clustered in one context, but not in the other. A differential co-expression score (DCS) can be derived from the differences in local posterior pairwise probabilities of gene co-expression between the two contexts. Given two contexts *c*_1 _and *c*_2_, we compute the gene-specific DCS as follows:

1) For each context *c*,

a. Compute the *N *× *N *posterior pairwise probability (PPP) matrix of any two genes being co-clustered within *c*

b. Construct a hierarchical tree *T*_*c *_by applying average linkage hierarchical clustering with the local PPP matrix as similarity measure

2) Calculate the *N *× *N *matrix **D**_*iff *_= (*d*)_*N*, *N *_= abs(PPP_*c*1_-PPP_*c*2_) of absolute differences between the two PPP matrices

3) For each context *c*,

a. Cut *T*_*c *_at all possible levels to obtain a list of gene clusters **G**_*c *_where cutting *T*_*c *_at level (1-*p*) induces a gene clustering such that the average PPP between each pair of genes within a resulting cluster is greater than *p*.

b. For each gene cluster *g *in **G**_*c*_

i. For each gene *i*, compute the score DCS_*cluster*_(*i*, *g*, *c*)

DCS_*cluster*_(*i*, *g*, *c*) = Σ*d*_*ij*_/(|*g*|-1), if genes *i*, *j *are in *g*, *i *≠ *j*, and |*g*| is size of cluster *g*.

DCS_*cluster*_(*i*, *g*, *c*) = 0, if *i *is not in *g*.

4) For each gene *i*, compute the gene-specific score DCS_*gene*_(*i*) = max_{*g*, *c*}_(DCS_*cluster*_(*i*, *g*, *c*))

### Simulation study

The simulation study was performed by generating random datasets with the clustering/context structure as depicted in Figures 1 at various levels of "noise". For each noise level, 100 random datasets were generated and analyzed. ROC curves and areas under the curves were calculated by averaging over all 100 random datasets for each scenario.

Each simulated *N *× *M *data matrix **X **comprises four gene clusters and three contexts. Clusters 1 and 2 each have 20 genes while clusters 3 and 4 each have 80 genes. Each of the three contexts has five samples. Thus, *M *= 15 and *N *= 200. Each gene expression profile *x*_*i *_is generated by one of four underlying patterns representing the four gene clusters such that *x*_*i *_~*N*(*μ*_*c*_, *σ*^2^), *μ*_*c *_= (*μ*_*c*1_, ..., *μ*_*cM*_) and gene *i *is generated by pattern *c*. For clusters 3 and 4, *μ*_*c *_is identical for all samples, that is "low" (= 0) and "high" (= 1), respectively. In contrast, for cluster 1, *μ*_*c *_is "high" for samples 1-5 and low for samples 6-15 while for cluster 2, *μ*_*c *_is "high" for samples 6-10. Thus, only gene clusters 1 and 2 are informative in distinguishing the three contexts. The noise parameter *σ *is the same for all clusters and context ranging from 0.4 to 0.8. Each simulation is repeated 100 times. Figure 1A shows a heatmap of one of the simulated datasets at the *σ *= 0.5 noise level. For the simulation scenario in Figure 1D, we modify the mean expression profiles so that the mean *μ*_*c *_is set to -1 instead of 1 for samples 1-2, 6-8, and 11-12, thus leaving the co-expression patterns (and contexts) intact but changing the expression levels in some samples.

### Breast cancer studies

#### Data preprocessing and gene selection

Raw data files for six human breast cancer datasets were RMA-preprocessed [52] separately using the Entrez Gene-based custom CDF (version 10) [53] and centered around their respective median. A mild variation filter using Cancer Outlier Profiler Analysis (COPA, 95^th ^percentile) [54] was applied to select the top 10,000 genes to be analyzed.

#### Survival analysis and other statistical analyses

Where multiple end points were available we chose disease-specific or metastasis-free survival rather than overall survival (Additional file 1; Table S4). Survival times were censored at 10 years. For the Cox regression analysis variables were dichotomized as follows. Tumor size: ≤/> 2 cm; tumor grade: grade 1/grades 2 and 3; ER status: +/-; AURKA gene expression (median): ≤/> median after preprocessing; AURKA gene expression (*k*-Means): cluster 1/cluster 2; computational methods: cluster 1/cluster 2.

Gene clusters were functionally analyzed using the *CLEAN *methodology [55], and the enrichment of DCE genes by estrogen regulated and oncogenic pathway genes was assessed using LRpath methodology [56]. Additional details are available in Additional file 1; Supplemental Methods.

## Authors' contributions

J.F. and M.M. conceived the project, developed the DCIM algorithm analyzed data and wrote the paper. S.S. assisted with the model development and developed the framework calculating posterior conditional distribution for assigning samples to contexts. M.W. assisted with the implementation of the computational algorithms and writing of the paper. All authors read and approved the final manuscript.

## Supplementary Material

Additional file 1 **Supplemental materials 1**. Word DOC containing Table S1-S6 and Figures S1-S4. Click here for file

Additional file 2 **Supplemental material 2**. XLS containing Table S7-S10. Click here for file
