# Supplementary material for: A semi-parametric Bayesian model for unsupervised differential co-expression analysis
Source: BMC Bioinformatics. 2010 May 7;11:234. doi: 10.1186/1471-2105-11-234 (PMC2876132; doi:10.1186/1471-2105-11-234)
Supplement: Additional file 1 — Supplemental materials 1. Word DOC containing Table S1-S6 and Figures S1-S4. [file 1471-2105-11-234-S1.DOC]

A semi-parametric Bayesian model for unsupervised differential co-expression analysis identifies novel molecular subtypes

Johannes Freudenberg, Siva Sivaganesan, Michael Wagner and Mario Medvedovic

Supplemental Tables

**Table S1:** Patient characteristics of DCIM derived patient groups for the Schmidt *et al.* dataset [1] as shown in Figure 3A (blue and green sample cluster, respectively).

| **Clinical Parameter** | **Odds ratio** | **One-sided Fisher *p*-value** |
| --- | --- | --- |
| **ER status** (ER+, ER-) | 9.33 | 7.0×10^-9^ |
| **Tumor size** (≤ 2cm, >2cm) | 1.97 | 1.8×10^-2^ |
| **Tumor grade** (G1, G2, G3) | (N/A) | 5.0×10^-9^ |

**Table S2:** Comparison of computationally derived patient groups for the Schmidt *et al.* dataset [1] showing the ratio of the number of patients placed in poor/favorable survival groups by both algorithms (concordant placements) divided by the number of all patients.

|  | **Pearson correlation** | **Euclidean distance** | **k-Means** | **DCIM** |
| --- | --- | --- | --- | --- |
| **Pearson correlation** | 1 | 0.735 | 0.935 | 0.74 |
| **Euclidean distance** | 0.735 | 1 | 0.77 | 0.765 |
| **k-Means** | 0.935 | 0.77 | 1 | 0.795 |
| **DCIM** | 0.74 | 0.765 | 0.795 | 1 |

**Table S3:** Predicting survival outcome using the top 200 DCE genes with different clustering algorithms (Schmidt *et al.* dataset [1]).

| **Gene List** | **Euclidean Distance** | | | **Pearson's Correlation** | | | **k-Means** | | |
| --- | --- | --- | --- | --- | --- | --- | --- | --- | --- |
|  | **size** | | **logrank *p*-value** | **size** | | **logrank *p*-value** | **size** | | **logrank p-value** |
|  | **group 1** | **group 2** |  | **group 1** | **group 2** |  | **group 1** | **group 2** |  |
| **Top 200 DCE genes** | 181 | 19 | 1.0×10^-1^ | 115 | 85 | 9.0×10^-1^ | 179 | 21 | 1.5×10^-2^ |

**Table S4:** Overview of the breast cancer studies used.

| **Reference** | [1] | [2] | [3] |
| --- | --- | --- | --- |
| **GEO accession** | GSE11121 | GSE3494 | GSE7390 |
| **Number of patients** | 200 | 251 | 198 |
| **Endpoint** | Metastasis-free survival | Disease-specific survival | Metastasis-free survival |
| **Treatment** | Surgery, 125 (63%) also received irradiation | Surgery, 110 (43%) patients also received adjuvant therapy | Surgery |
| **ER status** (+/-/NA) | 162/38/- | 213/34/4 | 134/34/- |
| **LN status** (+/-/NA) | -/200/- | 84/158/9 | -/198/- |
| **Tumor grade** (1/2/3/NA) | 29/136/35/- | 67/128/54/2 | 30/83/83/2 |
| **Age** (years)**: mean (standard deviation)** | 60 (12) | 62 (14) | 46 (7) |
| **Tumor size** (cm)**:** **mean (standard deviation)** | 2.1 (1.0) | 2.2 (1.3) | 2.2 (0.8) |

**Table S5:** Comparison of patient groupings derived from individual and joint (“meta”-) analyses.

|  | | **Individual analyses** | |
| --- | --- | --- | --- |
|  |  | **Favorable survival** | **Poor survival** |
| **Joint analysis** | **Favorable survival** | 494 (49.9%) | 65 (6.6%) |
|  | **Poor survival** | 139 (14.1%) | 291 (29.4%) |

**Table S6:** Overlap of the 500 DCS signature with other well-known breast cancer signatures.

| **Gene List** | **Reference** | **OR** | **Fisher p** |
| --- | --- | --- | --- |
| **Tumor subtypes  (“Intrinsic breast cancer genes”)** | Hu *et al.*, 2006,  BMC Bioinformatics [4] | 3.96 | 6.38E-30 |
| **Histologic grade** | Sotiriou *et al.*, 2006,  J Natl Cancer Inst. [5] | 2.49 | 2.23E-03 |
| **Clinical outcome  (“70-genes signature”)** | van't Veer *et al.*, 2002,  Nature [6] | 3.75 | 8.45E-03 |
| **Metastasis  (“76-genes signature”)** | Wang *et al.*, 2005,  The Lancet [7] | 2.49 | 7.96E-01 |

Supplemental Figures

**Figure S1. Analysis of bovine model data [8].** We downloaded the preprocessed data for the 11,057 genes and 10 developmental time points comparing wild type cross (Wagyu × Hereford) and double muscle cross (Piedmontese × Hereford) for a total of twenty data vectors (replicates were already averaged as part of the preprocessing). We then performed per-gene normalization as described and applied our DCIM model fit using all genes. Here, we are showing the heatmap and gene and sample clusterings for a subset defined in the original publication containing 85 differentially expressed (DE) genes and 920 transcription factors (panel A). In both cases, the resulting two top level sample contexts split into pre-natal and post-natal time points (panel B). In contrast, other hierarchical clustering methods (Euclidean distance (panel C), Pearson correlation (panel D)) consistently grouped the same time points (e.g. Piedmontese (P) and Wagyu (W) cattle at 280 days (280) or 3 months (03m)) as pairs of most similar samples but, unlike DCIM, created no obvious separation of time points. We then computed the differential co-expression score for Piedmontese vs. Wagyu cattle and applied Fisher’s test to compare the 85 DE genes and the top DCE genes. The resulting *p*-values were 1.6×10^-12^ and 6.7×10^-20^ for the top 85 and top 200 DCE genes, respectively. All but one DE gene (CYP4B1) had above median DCE scores. Myostatin (GDF8) ranked 432 in this analysis among all genes and ranked 30 among the 920 transcription factors identified by Hudson *et al*. We then repeated the functional analysis [9] for all genes ranked by DCS and found similar significantly enriched categories related to muscle structural components (such as “extracellular region”, “myofibril”, and “contractile fiber part”). The top GO term was “response to organic substance” (*p*-value 9.8×10^-12^) and after adjusting the data for the “time point effect”, top category was “extracellular region” (*p*-value 4.6×10^-12^).

**Figure S2. Cluster analysis results of two simulated data sets using traditional two-way clustering algorithms, bi-clustering methods, and the DCIM algorithm.** Data sets were generated following the simple simulation procedure (**a, b**) and the two fold changes scenario (**c, d**) as described with the low noise level σ=0.3 and mean expression levels *μ_c_* = 1, and -1, respectively, in the first scenario and *μ_c_* = 1, -1, and -2, respectively, in the second scenario. Cluster analysis was performed for both datasets using traditional two-way clustering methods and the DCIM algorithm (**a, c**), as well as the bi-clustering methods implemented in the Biclustering Analysis Toolbox v2.2 [10] showing representative subsets of the resulting consecutively numbered bi-clusters (**b, d**). In the simple scenario, the two-way clustering methods and DCIM easily recover the imposed contexts and in the case of DCIM and Euclidean distance hierarchical clustering the gene clusters as well (**a**). Somewhat surprisingly, none of the bi-clustering methods recovered the “ideal” bi-clusters indicated in panel (**b**) with only a few exceptions (e.g. BiMax #2). In the two fold changes scenario, only DCIM groups samples based on gene co-expression patterns thus correctly recovering the imposed contexts, while the two-way clustering methods group samples by relative expression levels (**c**). The bi-clustering methods again largely fail to recover the expected bi-clusters in this scenario (**d**).

**
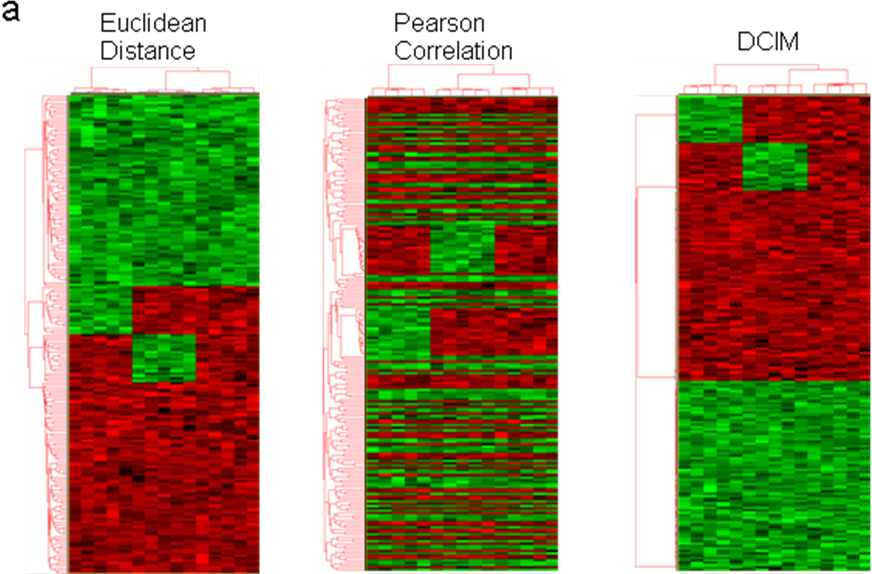
**

**
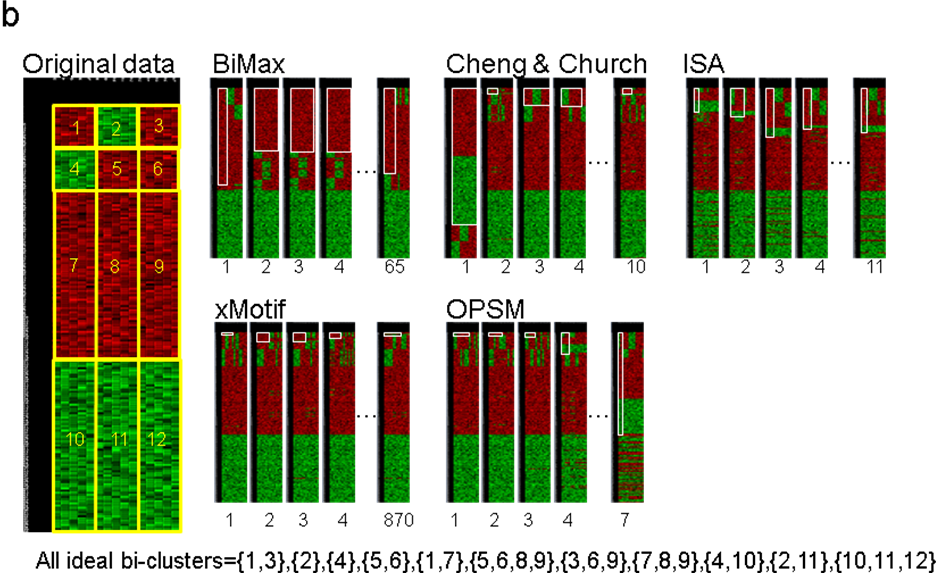
**

**
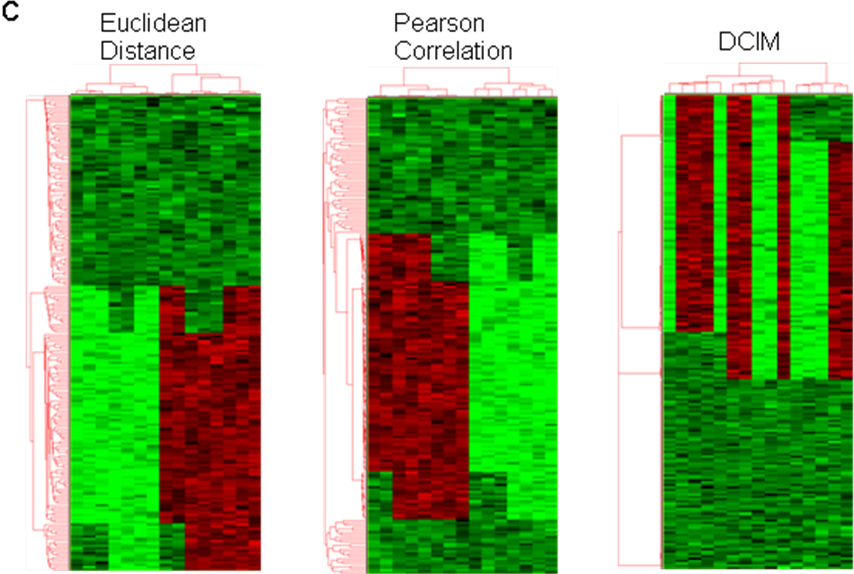
**

**
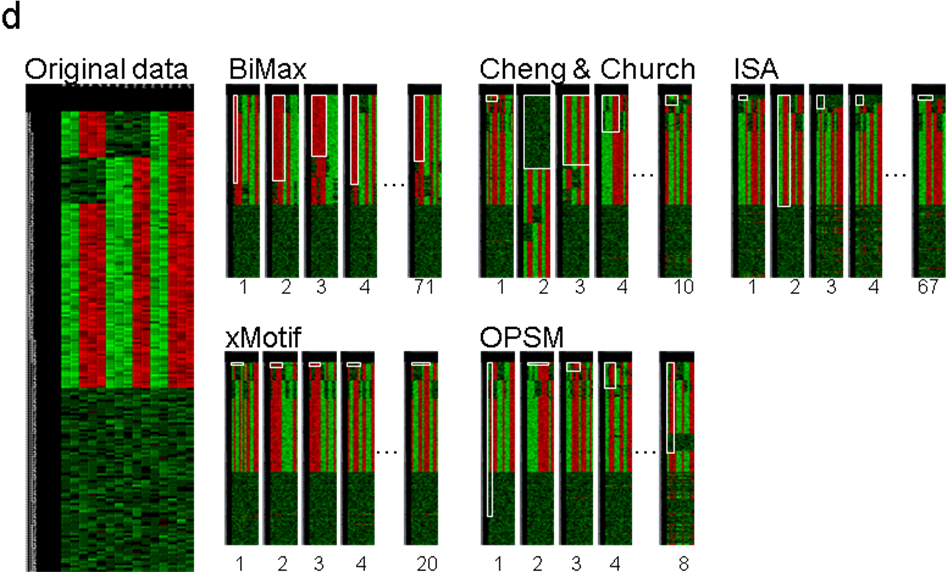
**

**Figure S3. Heatmaps of top 200 DCS gene signature (from Figure 3A) shown in three breast cancer studies.** We chose the top 200 DCE genes from the Schmidt *et al.* dataset and, similar to Figure 6, reanalyzed the three breast cancer datasets using only these genes. Expression patterns are remarkably consistent across different datasets. DCIM sample clusterings are highly correlated with ER status. Grey lines indicate that the corresponding gene was missing in the respective dataset after preprocessing.

**Figure S4. Cox regression survival analysis for different clinical/molecular parameters and their combinations.** Model fit was assessed in terms of Akaike Information Criterion (AIC). The lowest AIC indicating the best fit was achieved for model with the differential co-expression context groupings (DCIM) and Lymph Node (LN) status.


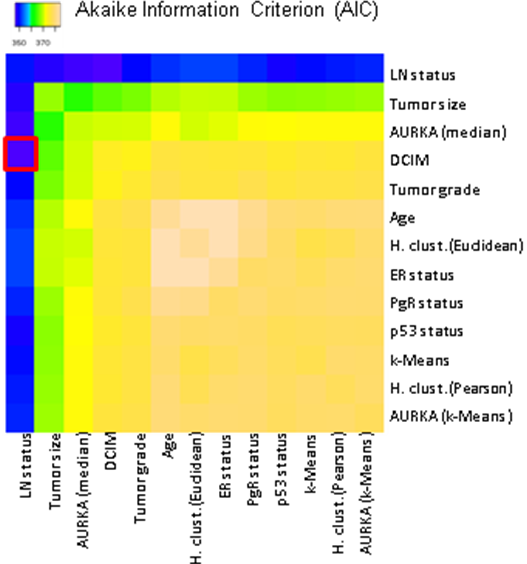


Supplemental Methods

**Infinite mixture model**

*Motivation*

In a typical application we have a set of gene expression signatures of biological samples which represent a range of different phenotypes. Examples include clinical parameters such as age, gender, tumor stage and tumor grade or molecular parameters such as estrogen receptor (ER) and progesterone receptor (PR) status. The different phenotypes likely correspond to a host of genetic pathways and other regulatory programs regulating the many functions of a cell within the particular tissue samples. Some of the activated pathways are expected to be common to all of the samples under investigation while others are likely to be specific to a particular subset of samples. Liu *et al.* show that explicitly accounting for different sample subtypes (or *contexts*) significantly improves gene clustering but their approach requires *a-priori* definition of contexts [11]. However, the optimal definition contexts based on clinical parameters is not always known or obvious but may, for example, correspond to molecular breast cancer subtypes [12,13]. We therefore here propose a method to infer *contexts* directly from the data.

Figure 1 illustrates the rationale for our Differential Co-expression Infinite Mixtures (DCIM) model. As in Liu *et al.* [11] we call a group of samples of the same subtype *context*. A group of genes co-expressed in all contexts is a *global cluster*, that is, these genes have a common expression pattern across all samples in the experiment. The common expression is assumed to be derived from a common underlying pattern. Global clusters that are indistinguishable locally, that is, within a given context, are grouped further into a *local cluster* of co-expressed genes. Conversely, groups of genes that are co-expressed (or co-clustered) only within specific subsets of samples but not across all samples thereby define contexts. As a result, each context is characterized by a unique gene co-clustering structure. To formally describe global and local gene clusters as well as contexts and their relation to the measured intensities we postulate the above mentioned Bayesian infinite mixture model.

*Model parameters*

Suppose we have a gene expression dataset for *N* genes and *M* biological samples or experimental conditions, **X** is the *N* × *M* expression matrix where *x_ij_* is the relative expression level of gene *i* in sample *j*. Accordingly, **x***_i_* = (*x_i1_*, *x_i2_*, …, *x_iM_*) is the expression profile for gene *I* and **x***_j_*^T^ = (*x_1j_*, *x_2j_*, …, *x_Nj_*)^T^ is the expression signature for sample *j*.

**x***_i_* is assumed to be generated by one out of *Q* underlying patterns. *C* = (*c*_1_, *c*_2_, …, *c_N_*)^T^ is the corresponding index variable; *c_i_* = *q* means that expression profile ***x****_i_* is generated by underlying pattern *q*, *q* = 1, …, *Q*, *Q* ≤ *N*. Patterns are represented by the *M*-variate normal distribution *Ν_M_*(*μ_q_*, *Σ_q_*), that is, *c_i_* = *q* implies **x***_i_* ~ *Ν_M_*(*μ_q_*, *Σ_q_*).

Likewise, samples within the same biological context are assumed to generate similar expression signatures. *D* = (*d*_1_, *d*_2_, …, *d_M_*) is an *M*-dimensional index variable *d_j_* = *r* means that expression signature **x***_j_*^T^ is assumed to originate from context *r* = 1, …, *R* ≤ *M* and each context *r* comprises 1 ≤ *M_r_* ≤ *M* samples or experimental conditions. The two extreme cases are *R* = 1 and *R* = *M*. The former means that all expression signatures originate from the same context and is equivalent to a simpler clustering model, where contexts are not defined. The latter means that each sample defines its own context.

Given *D* and a gene expression profile **x***_i_*, *R* subprofiles **x***_i_^1^*, **x***_i_^2^*, …, **x***_i_^R^* are defined such that **x***_i_^r^* = (*x_ij_r_(1)_*, *x_ij_r_(2)_*, …, *x_ij_r_(M_r)*) and *d_j_* = *r* for expression signatures *j_r_(1)*, *j_r_(2), …, j_r_(M_r_)*. In other words, the *R* contexts define *R* subprofiles of a given gene expression profile.

Each expression profile is grouped into one of *Q* clusters representing the underlying overall patterns. We term this the *global gene clustering*. Thus, each subprofile of an expression profile is implicitly grouped into one of *Q* global clusters. Locally however, that is, within a given context, groups of subprofiles may be indistinguishable and therefore form local groups of subprofiles termed *local clusters*. In other words, global gene clusters are grouped further into local gene clusters depending on the biological context of the sample. The local gene clustering structure is represented by a *Q* × *R* matrix **L** where *l_qr_* = *t* means that, within context *r*, global cluster *q* is grouped into local cluster *t*.

*Model specification*

To specify the distribution of the expression data as well as the parameters described above we postulate a Bayesian hierarchical model [14] displayed as a Directed Acyclic Graph (DAG) [15] in Figure 2. Here, nodes represent random variables and edges between the nodes indicate dependencies between nodes such that each random variable is conditionally independent of its non-descendents given its parent nodes (local Markov property). The joint probability distribution of the random variables is given by

$$\begin{matrix} p(X,C,D,L,M,M^{*},\Sigma,\alpha,\beta,\phi,a,\text{a},\lambda,\tau)=p(X|C,M,\Sigma)p(C|\alpha)p(M|L,D,M^{*}) \\ p(\Sigma|\beta,\phi)p(L|a)p(D|\text{a})p\text{(}M^{*}|\lambda,\tau) \\ p(\alpha)p(a)p(\lambda)p(\tau)p(\text{a)}p\text{(}\beta)p(\phi) \end{matrix}$$

where $M=\{\mu_{1},...,\mu_{Q}\}$ and $\Sigma=\{\Sigma_{1},...,\Sigma_{Q}\}$ are the mean vectors and variance-covariance matrices defining the expression patterns $x_{i}$, that is, $p(x_{i}|c_{i}=q,M,\Sigma)=f_{N}(x_{i}|\mu_{q},\Sigma_{q})$, $f_{N}(\cdot|\mu,\Sigma)$ is the multivariate normal distribution function. Distributions for the random variables defining the global gene clustering **C**, local gene clustering **L**, and sample to context assignment **D** each are defined according to the infinite mixture paradigm that does not require *a priori* specification of the number of clusters and contexts, respectively [11,16]. For example, the prior probability that a sample *j* will be placed in already existing context *r* is $p(d_{j}=r|D,\text{a})=\frac{n_{-j,r}}{M-1+\text{a}}$ while the prior probability of *j* being placed in a new context is $p(d_{j}\neq d_{j'},j\neq j'|D,\text{a})=\frac{\text{a}}{M-1+\text{a}}$ where $n_{-j,r}$ is the number of samples currently in context *r* without *j*. Probability distributions for the other parameters have been described previously [11] and are listed below.

**Variables in the model:**

**x***_i_*=(*x_i1_, x_i2_,…, x_iM_*) , *i*=1,…,*N* observed gene expression profiles for all *N* genes

**μ***_q_*=(*μ_q1_*,…, *μ_qM_*), *q*=1,…,*Q* the mean profile for global cluster *q*

**x***_i_^r^*=(*x_ij_r_(1)_*, *x_ij_r_(2)_*, …, *x_ij_r_(M_r)*) where *d_j_* = *r, r*=1,…,*R* is the expression profile for gene *i* within context *r*, *i*=1,…,*Q*

$\mu_{\text{tr}}^{\text{*}}$, mean expression profile for the local cluster *t* within context *r*

**M**=(**μ**_1_,…,**μ***_Q_*)

**Σ**=(**Σ**_1_,…,**Σ**_Q_), where each **Σ***_q_* is a diagonal matrix with context-specific cluster variances on the diagonal, that is **Σ**_q_=diag($\text{(σ}_{\text{q1}}^{2},....,\text{σ}_{\text{q1}}^{2}),\text{(σ}_{\text{q2}}^{2},....,\text{σ}_{\text{q2}}^{2}),...,\text{(σ}_{\text{qR}}^{2},....,\text{σ}_{\text{qR}}^{2})$)

**M**^*^=$\text{[(}\mu_{\text{1 }1}^{\text{*}},....,\mu_{\text{Q}_{1}1}^{\text{*}}),...,(\mu_{\text{1 R}}^{\text{*}},....,\mu_{\text{Q}_{\text{R}}\text{R}}^{\text{*}})]$

Hyperparameters λ**, τ**, **β** and **φ** are all assumed to be context-specific: **τ=(**τ_1_,…,τ_R_), **β=(**β_1_,…, β_R_), **φ=(**φ_1_,…, φ_R_); λ=(λ*^1^*,…,λ*^R^*), λ*^r^* = (*λ^r^_1_,…,λ^r^_Mr_*) where *M_r_* is the number of samples within context *r*.

**Prior Distribution of the Parameters:**

$$p(\text{ }x_{\text{i}}\text{ }|\text{c}_{\text{i}}=\text{q, }M\text{,}\Sigma)=f_{N}(\text{ }x_{\text{i}}\text{ }|\text{ }\mu_{\text{q}}\text{,}\Sigma_{\text{q}})$$

$$p(\text{ (}\mu_{\text{q1}}\text{,}\mu_{\text{q2}}\text{,...,}\mu_{\text{qR}}\text{) }|\text{ }L\text{, }M^{*}\text{,}\Sigma)=(f_{N}(\mu_{\text{q1}}\text{ }|\text{ }\mu_{\text{L}_{\text{q1}}1}^{*}\text{,}\Sigma_{1}),f_{N}(\mu_{\text{q2}}\text{ }|\text{ }\mu_{\text{L}_{\text{q2}}2}^{*}\text{,}\Sigma_{2}),...,f_{N}(\mu_{\text{qR}}\text{ }|\text{ }\mu_{\text{L}_{\text{qR}}\text{R}}^{*}\text{,}\Sigma_{\text{R}})$$

$p(\text{ c}_{\text{i}}=\text{q }|\text{ }C_{-\text{i}}\text{, }\alpha\text{)}\propto\frac{\text{n}_{-\text{i,q}}}{\text{N-1}+\text{α}}$**,** *q*=1,…,*Q*, $p(\text{ c}_{\text{i}}\neq\text{c}_{\text{i'}}\text{, i'}\neq\text{i }|\text{ }C_{-\text{i}}\text{, }\alpha\text{)}\propto\frac{\text{α}}{\text{T-1}+\text{α}}$**,** *i*=1,…,*N*, where *n*_-i,q_ is the number of profiles placed in global cluster *q* not counting the profile *i*

$p(\text{L}_{\text{qr}}=\text{t}|\text{C},\text{a})\text{ }\propto\text{ }\frac{\text{n}_{\text{-qrt}}}{\text{Q-1}+\text{a}}$, *t*=1,..,Q, $p(\text{L}_{\text{kf}}\neq\text{L}_{\text{k'f}},\forall\text{k'}\neq\text{k}|\text{C},\text{a})\text{ }\propto\frac{\text{a}}{\text{Q-1}+\text{a}}$ where *n_-qrt_* is the number of global clusters currently placed in local cluster *t* within context *r* without counting the *q*^th^ global cluster

$p(\mu_{\text{tr}}^{\text{*}}|\lambda_{\text{r}}\text{,τ}_{\text{r}})=f_{N}(\mu_{\text{tr}}^{\text{*}}|\lambda_{\text{r}},\text{τ}_{\text{r}}^{\text{-1}}I)$ $p(\text{σ}_{\text{tr}}^{\text{-2}}|\text{β}_{\text{r}}\text{,φ}_{\text{r}})=f_{G}(\text{σ}_{\text{tr}}^{\text{-2}}|\frac{\text{β}_{\text{tr}}}{2},\frac{\text{β}_{\text{r}}\text{φ}_{\text{r}}}{2})$, *r*=1,…,*R*

$$p(\text{φ}_{\text{r}}\text{|σ}_{\text{xr}}^{2})=f_{G}(\text{φ}_{\text{r}}|\frac{1}{2},\frac{\text{σ}_{\text{xr}}^{\text{-2}}}{2})$$

$$p(\text{β}_{\text{r}})=f_{G}(\text{β}_{\text{r}}|\frac{1}{2},\frac{1}{2})$$

$$p(\text{τ}_{\text{r}}\text{|σ}_{xr}^{2})=f_{G}(\text{τ}_{\text{r}}|\frac{1}{2},\frac{\text{σ}_{xr}^{2}}{2})$$

$$p(\lambda_{\text{r}}|\mu_{\text{xr}},\text{σ}_{\text{xr}}^{2})=f_{N}(\lambda_{\text{r}}|\mu_{\text{xr}},\text{σ}_{\text{xr}}^{2}I)$$

$$p(\text{α}^{\text{-1}})=f_{G}(\text{α}^{\text{-1}}|\frac{1}{2},\frac{1}{2})$$

where

$$\mu_{\text{xr}}=\frac{\sum_{\text{i}=1}^{\text{N}} x_{\text{i}}^{\text{r}}}{\text{N }}$$

$$\text{σ}_{\text{xr}}^{2}=\frac{\sum_{\text{i}=1}^{\text{N}} (x_{\text{i}}^{\text{r}}-\mu_{\text{xr}})`(x_{\text{i}}^{\text{r}}-\mu_{\text{xr}})}{\text{N n}_{\text{r}}\text{-1}}$$

**Conditional Posterior Distributions:**

, where $\bar{x}_{\text{tr}\bullet}=\frac{\sum_{\text{L}_{\text{c}_{\text{i}}\text{r}}=\text{t}} x_{\text{i}}^{\text{r}}}{\text{n}_{\text{tr}}^{\text{*}}}$ and $\text{n}_{\text{tr}}^{\text{*}}$ is the total number of expression profiles grouped in global clusters which are place in the local cluster *t* within the context *r*. Similarly, the variance for all global clusters place in the local cluster *t* within the context *r* is

$p(\text{ σ}_{\text{tr}}^{\text{-2}}\text{ }|\text{ }X,\text{ }M,\text{β}_{\text{r}}\text{,φ}_{\text{r}})\text{ }=\text{ }f_{G}(\text{ σ}_{\text{tr}}^{\text{-2}}\text{ }|\text{ }\frac{\text{r}_{\text{f}}\text{n}_{\text{tr}}^{\text{*}}+\text{β}_{\text{r}}}{2}\text{,}\frac{s_{\text{tr}}^{2}+\text{β}_{\text{r}}\text{φ}_{\text{r}}}{2})$, where $s_{\text{tr}}^{2}=\sum_{\text{L}_{\text{c}_{\text{i}}\text{r}}=\text{t}} \text{(}x_{\text{i}}^{\text{r}}-\mu_{\text{tr}}^{\text{*}})`\text{(}x_{\text{i}}^{\text{r}}-\mu_{\text{tr}}^{\text{*}})$

$$f(\text{ }\lambda_{\text{r}}\text{ }|\text{ }\mu_{\text{1r}}^{*}\text{,...,}\mu_{\text{Q}_{r}\text{r}}^{*}\text{,τ}_{\text{r}})\text{ }=\text{ }f_{N}(\text{ }\lambda_{\text{r}}\text{ }|\text{ }\frac{\text{σ}_{\text{xr}}^{2}\frac{\sum_{\text{t}=1}^{\text{K}_{\text{f}}} \mu_{\text{tr}}^{*}}{\text{Q}_{\text{r}}}+\frac{\text{τ}_{\text{r}}^{-1}}{\text{Q}_{\text{r}}}\mu_{\text{xr}}}{\text{σ}_{\text{x}}^{2}+\frac{\tau^{\text{-1}}}{\text{Q}}}\text{,}\frac{\text{σ}_{\text{xr}}^{2}\frac{\text{τ}_{\text{r}}^{-1}}{\text{Q}_{\text{r}}}}{\text{σ}_{\text{xr}}^{2}+\frac{\text{τ}_{\text{r}}^{-1}}{\text{Q}_{\text{r}}}}I)$$

$$f(\text{ τ}_{\text{r}}^{-1}\text{ }|\text{ }\mu_{\text{1r}}^{\text{*}}\text{,...,}\mu_{\text{Q}_{\text{r}}\text{r}}^{\text{*}},\lambda_{\text{r}})\text{ }=\text{ }f_{G}(\text{ τ}_{\text{r}}^{-1}\text{ }|\text{ }\frac{\text{n}_{\text{r}}\text{Q}_{\text{r}}+1}{2}\text{,}\frac{\sum_{\text{t}=1}^{\text{Q}_{\text{r}}} \text{(}\mu_{\text{tr}}^{\text{*}}\text{-}\lambda_{\text{r}}\text{)`(}\mu_{\text{tr}}^{\text{*}}\text{-}\lambda_{\text{r}}\text{)}+\text{σ}_{ \text{xr}}^{2}}{2})$$

$$f(\text{ φ}_{\text{r}}\text{ }|\text{ σ}_{\text{1r}}^{\text{-2}}\text{,...,σ}_{\text{Q}_{\text{r}}\text{r}}^{\text{-2}}\text{,β}_{\text{r}})=\text{ }f_{G}(\text{φ}_{\text{r}}\text{ | }\frac{\text{Q}_{\text{r}}\text{β}_{\text{r}}+1}{2}\text{ , }\frac{\text{β}_{\text{r}}\sum_{\text{t}=1}^{\text{Q}_{\text{r}}} \text{σ}_{\text{tr}}^{\text{-2}}+\text{σ}_{\text{xr}}^{\text{-2}}}{2})$$

$$f(\text{ β}_{\text{r}}\text{ }|\text{ σ}_{\text{tr}}^{\text{-2}}\text{,...,σ}_{\text{tr}}^{\text{-2}}\text{,φ}_{\text{r}})\text{ }=\text{ }\alpha\text{ }\Gamma(\frac{\text{β}_{\text{r}}}{2})(\frac{\text{β}_{\text{r}}}{2})^{\frac{\text{(Q}_{\text{r}}\text{β}_{\text{r}}\text{-3)}}{2}}\text{exp}\left(-\frac{\text{β}_{\text{r}}^{\text{-1}}\text{ }}{2}\right)\prod_{\text{t}=1}^{\text{Q}_{\text{r}}} \left[\text{(φ}_{\text{r}}\text{σ}_{\text{tr}}^{\text{-2}}\text{)}^{\frac{\text{β}_{\text{r}}}{2}}\text{ exp}\left(\text{-}\frac{\text{σ}_{\text{tr}}^{\text{-2}}\text{φ}_{\text{r}}\text{β}_{\text{r}})}{2}\right)\text{ }\right]$$

$$f(\text{ α }|\text{ Q,N})\text{ }=\alpha\text{ }\frac{\text{α}^{\text{Q-}\frac{3}{2}}\exp(-\frac{1}{2\text{α}})\Gamma(\text{α})}{\Gamma(\text{N}+\text{α})}$$

$p(\text{ c}_{\text{i}}=\text{q }|\text{ }C_{-\text{i}}\text{, }L\text{,}x_{\text{i}}\text{,}M\text{,}\Sigma\text{)}\propto\frac{\text{n}_{-\text{i,q}}}{\text{N-1}+\text{α}}\text{ }f_{N}(\text{ }x_{\text{i}}\text{ }|\text{ }\mu_{\text{q}}\text{,}\Sigma_{\text{q}}\text{)}$

$p(\text{ c}_{\text{i}}\neq\text{c}_{\text{i'}}\text{, i'}\neq\text{i }|\text{ }C_{-\text{i}}\text{, }x_{\text{i}}\text{, }\mu_{x}\text{,σ}_{x}^{2}\text{)}\propto\frac{\text{α}}{\text{N-1}+\text{α}}\left(\left[\int f_{N}(\text{ }x_{\text{i}}\text{ }|\text{ }\mu_{\text{q}}\text{,}\Sigma_{\text{q}}\text{) }p(\mu_{\text{q}}\text{,}\Sigma_{\text{q}}|\lambda\text{,}\tau)\text{ d}\mu_{\text{q}}\text{d}\Sigma_{\text{q}}\right]\right)$

$$p(\text{L}_{\text{qr}}=\text{t}|X,\text{a})\text{ }\propto\text{ }\frac{\text{n}_{\text{-qrt}}}{\text{Q-1}+\text{a}}f_{N}(\text{ }\bar{x}_{\text{f}}^{\text{q}}|\mu_{\text{tr}}^{*},\frac{\text{σ}_{\text{tr}}^{2}}{\text{n}_{\text{q}}}I\text{)}$$

, where *n_-i,q_* is the number of profiles in global cluster *q* without counting profile *i*, and *n_-qrt_* is the number of global clusters grouped into local cluster *t* within context *r* not counting *q*^th^ global cluster, and $\bar{x}_{\text{r}}^{\text{q}}=\frac{\sum_{\text{c}_{\text{i}}=\text{q}} x_{\text{i}}^{\text{r}}}{\text{n}_{\text{q}}}$.

*Model fit*

Given data, that is, a set of gene expression profiles, the goal is to estimate the posterior distribution of the model parameters $p(C,D,L,M,M^{*},\Sigma,\alpha,\beta,\phi,a,\text{a},\lambda,\tau| X)$ and in particular, the marginal distribution of parameters **C**, **L**, and **D** given the data, which describe the gene clusterings and the contexts.

We accomplish this goal by implementing a Gibbs sampler [17], a Markov chain Monte Carlo algorithm that can be used when the conditional probability for each random variable in the model is known given all other variables. Our implementation is an extension of the previously described algorithm [11]. In particular, we use the following posterior conditional probability distribution to update contexts ***D***, that is, to place sample *j* into existing context *r*:

$$p(\text{ d}_{j}=r\text{ }|\text{ }x_{j}\text{,}D_{-j}\text{, L,}\Sigma,\lambda\text{,}\tau\text{)}\propto\frac{n_{-jr}}{M-1+a}\prod_{q=1}^{Q_{r}} \int\prod_{\{i|c_{i}=t,L_{tr}=q\}} f_{N}(x_{j}^{i}|\mu_{jrq},\Sigma_{rq}\text{)}p(\mu_{jrq}\text{,}\Sigma_{rq}|\lambda\text{,}\tau)d\mu_{jrq}$$

$$p(\text{ d}_{j}\neq\text{d}_{j'},j\neq j'\text{ }|\text{ }x_{j}\text{,}D_{-j}\text{, C, }\Sigma,\lambda\text{,}\tau\text{)}\propto\text{ }\frac{a}{M-1+a}\prod_{q=1}^{Q} \int\prod_{\{i|c_{i}=q\}} f_{N}(x_{j}^{i}|\mu_{jq},\Sigma_{q}\text{)}p(\mu_{jq}\text{,}\Sigma_{q}|\lambda\text{,}\tau)d\mu_{jq}$$

Notice that we integrate out the parameter $\mu_{q}$in the likelihood function. As a result, the posterior probability function does not depend on the mean vectors but it accounts for the specific local gene clusterings **L**, that is, the better a local clustering *L_r_* fits sample *j* the higher the likelihood.

All other conditional posterior distribution functions are as described in [11] and reproduced above. We also adapt the “reverse annealing” procedure [11,18] to counteract situations where the Gibbs samples do not “mix” well.

To summarize the Gibbs samples of clustering variable **C** and the context variable **D** we use the previously described approach of first averaging clusterings thus computing posterior pairwise probabilities (PPPs) and then generating a hierarchical clustering with the PPPs as the similarity measure and average linkage as the agglomeration strategy [11,16].

We extend this approach to compute “local” or context-specific PPPs. Let *S* be a context, *S* = {sample*_j_*}. After each Gibbs step *g*, we have recorded the parameters *C*(*g*), *D*(*g*), and **L**(*g*). Given *g*, we can now generate a sample-specific clustering *C_s_*(*g*) := (*c*(*_s_*_)1_(*g*), *c*(*_s_*_)2_(*g*), …, *c*(*_s_*)*_N_*(*g*))^T^, for all *s* in *S*, where

- - *C_s_*(*g*) is an index variable. *c*(*_s_*)*_i_* = *q*(*_s_*) means, that, after Gibbs step *g*, for sample *s*, gene *i* was assigned to sample-specific cluster *q*(*_s_*)
  - *d_s_*(*g*) = *r* (i.e., “sample *s* was in context *r* after Gibbs step *g*”),
  - *l_qr_*(*g*) = *t* (i.e., “within context *r*, global cluster *q* was grouped into local cluster *t* after Gibbs step *g*”)
  - *c_i_*(*g*)= *q* (i.e., “gene *i* was assigned to global cluster *q* after Gibbs step *g*”)
  - *c*(*_s_*)*_i_*(*g*) := *t*

In other words, the sample-specific local clustering after each Gibbs step is identical to the local clustering corresponding to the context the sample was a member of after that Gibbs step. *C_s_*(*g*) is generated for all samples *s* in *S* and for all Gibbs steps after the “burn-in” phase. Local PPPs specific to context *S* can now be computed for all gene pairs using the same procedure as for the global gene PPPs.

*Availability*The above described algorithms are available as part of the R package gimmR which can be downloaded from the authors’ website (http://Clusteranalysis.org/). The software generates output files that can be viewed and analyzed both directly in R [19] and using the FTreeView program [20].

**Differential co-expression score**

As described above, our model describes groups of samples or contexts and both global and local, that is context-specific, gene clusterings. Given this framework and given two contexts, we consider a pair of genes differentially co-expressed (DCE), if they are co-clustered in one context, but not in the other. Contexts are user-defined but this choice is typically guided by the posterior distribution of the **D** parameter in the model.

The definition of co-expression depends on pairs if not larger groups of genes, but it does not depend on groupings of samples. Gene co-expression groups (i.e. clusters) can be viewed by the user with tools such as TreeView [21] which also facilitate further analysis such as determining functional enrichment of gene clusters [20]. Differential expression refers to single genes but depends on at least two groups of samples. In this case, researchers are accustomed to assigning a score (fold change, *p*-value, etc.) to each gene allowing them to rank and prioritize genes. The challenge with the analysis of differential co-expression lies in the fact that it depends on groupings of both, genes and biological samples. Simultaneously viewing clusterings for two contexts would be too complex for all practical purposes as would be an attempt to prioritize pairs of genes based on a score. Therefore, we here propose a gene-specific differential co-expression score (DCS) which assigns a single number to each gene while at the same time accounting for the context-specific clusterings of the genes. The intuition being that the majority of genes typically will remain co-clustered with the same group of genes regardless of context. A smaller number of genes, however, are expected to have changing co-expression patterns. This behavior is reflected in the differences of pairwise gene distances for the context-specific clusterings. The score will be computed by averaging these differences for each gene, not over all possible gene pairs but over only the pairs within a local gene cluster within either context.

Given two contexts *c_1_* and *c_2_*, we compute the gene-specific DCS as follows:

1. For each context *c*,
   1. Compute the *N×N* posterior pairwise probability (PPP) matrix of any two genes being co-clustered within *c*
   2. Construct the hierarchical tree *T_c_* by applying average linkage hierarchical clustering with the local PPP matrix as similarity measure
2. Calculate the *N×N* matrix **D***_iff_* =(*d*)*_N,N_* = abs(PPP*_c1_*-PPP*_c2_*) of absolute differences between the two PPP matrices
3. For each context *c*,
   1. Cut *T_c_* at all possible levels to obtain a list of gene clusters **G***_c_* where cutting *T_c_* at level (1‑*p*) induces a gene clustering such that the average PPP between each pair of genes within a resulting cluster is greater than *p*.
   2. For each gene cluster *g* in **G***_c_*
      1. For each gene *i*, compute the score DCS*_cluster_*(*i*, *g*, *c*)
         DCS*_cluster_*(*i*, *g*, *c*) = Σ*d_ij_*/(|*g*|-1), if genes *i*,*j* are in *g*, *i*≠*j*, and |*g*| is size of cluster *g*.
         DCS*_cluster_*(*i*, *g*, *c*) = 0, if *i* is not in *g.*
4. For each gene *i*, compute the gene-specific score DCS*_gene_*(*i*) = max{*_g,c_*}(DCS*_cluster_*(*i*, *g*, *c*))

**Simulation study**

We designed a simple data simulation procedure to study the ability of different algorithms to correctly identify gene clusters and sample clusters or contexts as previously described [11]. As in the example shown in Figure 1, each simulated *N*×*M* data matrix **X** comprises four gene clusters and three contexts. Clusters 1 and 2 each have 20 genes while clusters 3 and 4 each have 80 genes. Each of the three contexts has five samples. Thus, *M*=15 and *N*=200. Each gene expression profile *x_i_* is assumed to be generated by one of four underlying patterns representing the four gene clusters such that *x_i_* ~ *N*(*μ_c_*, *σ^2^*), *μ_c_* = (*μ_c1_, …, μ_cM_*) and gene *i* is generated by pattern *c*. For clusters 3 and 4, *μ_c_* is assumed to be identical for all samples, that is “low” (=0) and “high” (=1), respectively. In contrast, for cluster 1, *μ_c_* is assumed to be “high” for samples 1-5 and low for samples 6-15 while for cluster 2, *μ_c_* is “high” for samples 6-10. Thus, only gene clusters 1 and 2 allow distinguishing the three contexts. The noise parameter *σ* is the same for all clusters and context ranging from 0.4 to 0.8. Each simulation is repeated 100 times. Figure 1 shows a heatmap of one of the simulated datasets at the σ=0.5 noise level. In addition, we slightly modify the procedure described above to generate a second set of simulations where the *μ_c_* values are set to -1 instead of 1 for samples 1-2, 6-8, and 11-12 thus leaving the co-expression patterns (and contexts) intact but changing the expression levels in some samples (see Figure 1D-F).

Given a hierarchical clustering of samples, we compute Receiver Operating Characteristics (ROC) based on the number of correctly or incorrectly co-clustered pairs of samples after cutting the tree at each possible distinct level (1 .. *M*). Given hierarchical tree *T* and level *p*, cutting *T* horizontally at level *p* induces a sample clustering with *N_p_* clusters, 1 ≤ *N_p_* ≤ *M*. For a given level *p*, we can therefore compute the true positive rate (TPR) and false positive rate (FPR) by assessing for each pair of co-clustered samples whether or not they both are in the same ‘true’ context. As a result, we have *M* FPR-TPR pairs, one for each cutting level, and can plot the ROC curve. We average ROC curves over multiple simulations by averaging the corresponding FPRs and TPRs at each distinct tree cutting level. Since the ROC curves are piecewise linear it is straightforward to compute the area under the curve (AUC).

Bi-clustering procedures are not designed to cluster all samples, thus we cannot construct equivalent ROC curves for these methods. Instead we show bi-clustering results for two exemplary, low-noise (σ=0.3) data sets, one for each simulation scenario. We use the Biclustering Analysis Toolbox (BiCAT) v2.2 [10] which provides implementations of BiMax [22], Cheng & Church’s algorithm [23], Iterative Signature Algorithm (ISA) [24,25], Order-preserving Submatrix Algorithm (OPSM) [26], and the xMotif algorithm [27]. We used the default parameter settings for the analysis of the two datasets as implemented in the BiCAT version 2.2 for Windows with the following exceptions. BiMax: Discretization percentage 53.33% and 28.67%, respectively (representing the respective proportions of simulated upregulated relative expression measures); Cheng & Church: δ=0.2; ISA: *t_g_*=*t_c_*=1.0; and xMotif: Maximum *p*=1×10^-4^.

## Breast cancer studies

## *Data preprocessing and gene selection*

Raw data files (Affymetrix HG-U133A, HG-U133+2.0 CEL files) of six human breast cancer datasets (GEO expression series GSE11121 [1], GSE1456 [28], GSE2990 [5], GSE3494 [2], GSE7390 [3], and GSE9195 [29] were downloaded from the public repository GEO [30]. Each dataset was RMA-preprocessed [31] separately using the Entrez Gene-based custom CDF (version 10) from the Psychiatry/MBNI Microarray Lab at the University of Michigan (‘Brainarray’) [32]. We applied a mild variation filter using Cancer Outlier Profiler Analysis (COPA, 95^th^ percentile) [33] to select the top 10,000 genes to be clustered in each of the human breast cancer datasets. In each dataset expression profiles were centered by setting the median expression value of each gene to zero (subtracting the gene-specific medians). Table 1 summarizes patient characteristics for these four independent datasets.

We created a joint expression data set from these six data sets as follows. First we identified unique patient samples based on the patient annotation made available on the GEO website. Next, we RMA-preprocessed and per-gene normalized all unique samples separately for each microarray platform (Affymetrix HG-U133A and HG-U133+2.0, respectively) again using the Entrez Gene-based custom CDF (version 10). Finally, we combined the resulting expression sets by matching all 11,961 Entrez Gene ID based probesets represented on both platforms.

## *Survival analysis and other statistical analyses*

We computed Kaplan-Meier curves and Cox proportional hazard regression using the *survival* package in R [19]. Survival times and end points together with other annotation data were obtained directly from the GEO website using the *GEOquery* R package. Where multiple end points were available we chose disease-specific or metastasis-free survival rather than overall survival (Table 1). The included studies differed considerably by observation time while typical endpoints in clinical trials are 5 year overall or disease-specific survival. To make results more comparable across studies we therefore censored the observation time at 5 and/or 10 years. For the Cox proportional hazard model fit, we dichotomized parameters as follows. Tumor size: ≤/> 2cm; tumor grade: grade 1/grades 2 and 3; ER status: +/-; AURKA gene expression (median): ≤/> median after preprocessing; AURKA gene expression (*k*-Means): cluster 1/cluster 2; computational methods: cluster 1/cluster 2.

All statistical analyses were performed using the statistical programming environment R version 2.7.1 [19] and Bioconductor release 2.2 [34]. This includes other clustering algorithms, namely hierarchical clustering with Euclidean distance or 1‑Pearson correlation as distance measure and *k*-Means clustering. We applied average linkage as the agglomeration strategy when DCIM’s 1‑PPP or 1‑Pearson correlation was the distance measure and complete linkage for Euclidean distance. Unless otherwise stated, *k*-Means implies *k*=2.

For functional annotation of gene lists and clusterings was done with the R package *CLEAN* [20] with functional categories derived from GO [35], KEGG [36], L2L [37], among other computationally and literature derived categories.

An R package containing the DCIM algorithm and the scripts used for our analysis are available at the supplemental website (<http://Clusteranalysis.org/>). Additional results clustering results using DCIM algorithm are available through Genomics Portals ([http://GenomicsPortals.org](http://GenomicsPortals.org/)) [38]

References

1. Schmidt M, Bohm D, von TC, Steiner E, Puhl A, Pilch H, Lehr HA, Hengstler JG, Kolbl H, Gehrmann M: The humoral immune system has a key prognostic impact in node-negative breast cancer. Cancer Res 2008, 68:5405-5413.

2. Miller LD, Smeds J, George J, Vega VB, Vergara L, Ploner A, Pawitan Y, Hall P, Klaar S, Liu ET et al.: From The Cover: An expression signature for p53 status in human breast cancer predicts mutation status, transcriptional effects, and patient survival. PNAS 2005, 102:13550-13555.

3. Desmedt C, Piette F, Loi S, Wang Y, Lallemand F, Haibe-Kains B, Viale G, Delorenzi M, Zhang Y, d'Assignies MS et al.: Strong time dependence of the 76-gene prognostic signature for node-negative breast cancer patients in the TRANSBIG multicenter independent validation series. Clin Cancer Res 2007, 13:3207-3214.

4. Hu Z, Fan C, Oh DS, Marron JS, He X, Qaqish BF, Livasy C, Carey LA, Reynolds E, Dressler L et al.: The molecular portraits of breast tumors are conserved across microarray platforms. BMC Genomics 2006, 7:96.

5. Sotiriou C, Wirapati P, Loi S, Harris A, Fox S, Smeds J, Nordgren H, Farmer P, Praz V, Haibe-Kains B et al.: Gene Expression Profiling in Breast Cancer: Understanding the Molecular Basis of Histologic Grade To Improve Prognosis. J Natl Cancer Inst 2006, 98:262-272.

6. van ', V, Dai H, van d, V, He YD, Hart AA, Mao M, Peterse HL, van der KK, Marton MJ, Witteveen AT et al.: Gene expression profiling predicts clinical outcome of breast cancer. Nature 2002, 415:530-536.

7. Wang Y, Klijn JG, Zhang Y, Sieuwerts AM, Look MP, Yang F, Talantov D, Timmermans M, Meijer-van Gelder ME, Yu J et al.: Gene-expression profiles to predict distant metastasis of lymph-node-negative primary breast cancer. Lancet 2005, 365:671-679.

8. Hudson NJ, Reverter A, Dalrymple BP: A differential wiring analysis of expression data correctly identifies the gene containing the causal mutation. PLoS Comput Biol 2009, 5:e1000382.

9. Eden E, Navon R, Steinfeld I, Lipson D, Yakhini Z: GOrilla: a tool for discovery and visualization of enriched GO terms in ranked gene lists. BMC Bioinformatics 2009, 10:48.

10. Barkow S, Bleuler S, Prelic A, Zimmermann P, Zitzler E: BicAT: a biclustering analysis toolbox. Bioinformatics 2006, 22:1282-1283.

11. Liu X, Sivaganesan S, Yeung KY, Guo J, Bumgarner RE, Medvedovic M: Context-specific infinite mixtures for clustering gene expression profiles across diverse microarray dataset. Bioinformatics 2006, 22:1737-1744.

12. Sorlie T, Perou CM, Tibshirani R, Aas T, Geisler S, Johnsen H, Hastie T, Eisen MB, van de RM, Jeffrey SS et al.: Gene expression patterns of breast carcinomas distinguish tumor subclasses with clinical implications. Proc Natl Acad Sci U S A 2001, 98:10869-10874.

13. Sorlie T, Tibshirani R, Parker J, Hastie T, Marron JS, Nobel A, Deng S, Johnsen H, Pesich R, Geisler S et al.: Repeated observation of breast tumor subtypes in independent gene expression data sets. Proc Natl Acad Sci U S A 2003, 100:8418-8423.

14. Gelman A, Carlin JC, Stern HS, Rubin DB: Bayesian Data Analysis. New York: CRC Press; 2003.

15. Cowell RG, Dawid PA, Lauritzen SL, Spiegelhalter DJ: Probabilistic Networks and Expert Systems. New York: Springer; 1999.

16. Medvedovic M, Sivaganesan S: Bayesian infinite mixture model based clustering of gene expression profiles. Bioinformatics 2002, 18:1194-1206.

17. Gelfand EA, Smith FMA: Sampling-based approaches to calculating marginal densities. Journal of the American Statistical Association 1990, 85:398-409.

18. Medvedovic M, Yeung KY, Bumgarner RE: Bayesian mixture model based clustering of replicated microarray data. Bioinformatics 2004, 20:1222-1232.

19. R Development Core Team: R: A Language and Environment for Statistical Computing. Vienna, Austria; 2008.

20. Freudenberg JM, Joshi VK, Hu Z, Medvedovic M: CLEAN: CLustering Enrichment ANalysis. BMC Bioinformatics 2009, 10:234.

21. Saldanha AJ: Java Treeview--extensible visualization of microarray data. Bioinformatics 2004, 20:3246-3248.

22. Prelic A, Bleuler S, Zimmermann P, Wille A, Buhlmann P, Gruissem W, Hennig L, Thiele L, Zitzler E: A systematic comparison and evaluation of biclustering methods for gene expression data. Bioinformatics 2006, 22:1122-1129.

23. Cheng Y, Church GM: Biclustering of expression data. Proc Int Conf Intell Syst Mol Biol 2000, 8:93-103.

24. Ihmels J, Bergmann S, Barkai N: Defining transcription modules using large-scale gene expression data. Bioinformatics 2004, 20:1993-2003.

25. Ihmels J, Friedlander G, Bergmann S, Sarig O, Ziv Y, Barkai N: Revealing modular organization in the yeast transcriptional network. Nat Genet 2002, 31:370-377.

26. Ben-Dor A, Chor B, Karp R, Yakhini Z: Discovering local structure in gene expression data: the order-preserving submatrix problem. J Comput Biol 2003, 10:373-384.

27. Murali TM, Kasif S: Extracting conserved gene expression motifs from gene expression data. Pac Symp Biocomput 2003,77-88.

28. Pawitan Y, Bjohle J, Amler L, Borg AL, Egyhazi S, Hall P, Han X, Holmberg L, Huang F, Klaar S et al.: Gene expression profiling spares early breast cancer patients from adjuvant therapy: derived and validated in two population-based cohorts. Breast Cancer Res 2005, 7:R953-R964.

29. Loi S, Haibe-Kains B, Desmedt C, Wirapati P, Lallemand F, Tutt AM, Gillet C, Ellis P, Ryder K, Reid JF et al.: Predicting prognosis using molecular profiling in estrogen receptor-positive breast cancer treated with tamoxifen. BMC Genomics 2008, 9:239.

30. Wheeler DL, Barrett T, Benson DA, Bryant SH, Canese K, Chetvernin V, Church DM, Dicuccio M, Edgar R, Federhen S et al.: Database resources of the National Center for Biotechnology Information. Nucleic Acids Res 2008, 36:D13-D21.

31. Bolstad BM, Irizarry RA, Astrand M, Speed TP: A comparison of normalization methods for high density oligonucleotide array data based on variance and bias. Bioinformatics 2003, 19:185-193.

32. Dai M, Wang P, Boyd AD, Kostov G, Athey B, Jones EG, Bunney WE, Myers RM, Speed TP, Akil H et al.: Evolving gene/transcript definitions significantly alter the interpretation of GeneChip data. Nucl Acids Res 2005, 33:e175.

33. Tomlins SA, Rhodes DR, Perner S, Dhanasekaran SM, Mehra R, Sun XW, Varambally S, Cao X, Tchinda J, Kuefer R et al.: Recurrent fusion of TMPRSS2 and ETS transcription factor genes in prostate cancer. Science 2005, 310:644-648.

34. Gentleman RC, Carey VJ, Bates DM, Bolstad B, Dettling M, Dudoit S, Ellis B, Gautier L, Ge Y, Gentry J et al.: Bioconductor: open software development for computational biology and bioinformatics. Genome Biol 2004, 5:R80.

35. Ashburner M, Ball CA, Blake JA, Botstein D, Butler H, Cherry JM, Davis AP, Dolinski K, Dwight SS, Eppig JT et al.: Gene ontology: tool for the unification of biology. The Gene Ontology Consortium. Nat Genet 2000, 25:25-29.

36. Kanehisa M, Goto S: KEGG: kyoto encyclopedia of genes and genomes. Nucleic Acids Res 2000, 28:27-30.

37. Newman JC, Weiner AM: L2L: a simple tool for discovering the hidden significance in microarray expression data. Genome Biol 2005, 6:R81.

38. Shinde K, Phatak M, Freudenberg JM, Chen J, Li Q, Joshi VK, Hu Z, Ghosh K, Meller J, Medvedovic M: Genomics Portals: integrative web-platform for mining genomics data. BMC Genomics 2010, 11:27.
